# Novel Bruton’s tyrosine kinase inhibitor TAS5315 suppresses the progression of inflammation and joint destruction in rodent collagen-induced arthritis

**DOI:** 10.1371/journal.pone.0282117

**Published:** 2023-02-23

**Authors:** Daichi Akasaka, Satoru Iguchi, Ryusuke Kaneko, Yohei Yoshiga, Daisuke Kajiwara, Yoshinori Nakachi, Naruto Noma, Kenji Tanaka, Atsushi Shimizu, Fumihito Hosoi

**Affiliations:** Discovery and Preclinical Research Division, Taiho Pharmaceutical Co., Ltd., Tsukuba, Ibaraki, Japan; University of Texas Southwestern Medical Center, UNITED STATES

## Abstract

Rheumatoid arthritis is an inflammatory autoimmune disease, characterized by autoantibody production, synovial inflammation, and joint destruction. Its pathogenesis is due to environmental factors and genetic backgrounds. Bruton’s tyrosine kinase is a cytoplasmic non-receptor tyrosine kinase, expressed in most hematopoietic cell lineages, except T cells and plasma cells, and regulates various immune-related signaling pathways, thereby playing a crucial role in pathogenesis. Thus, inhibiting Bruton’s tyrosine kinase may prove beneficial in treating autoimmune diseases. In the present study, we characterized Bruton’s tyrosine kinase inhibitor, TAS5315, *in vitro* and evaluated its therapeutic effects in experimental arthritis models. TAS5315 markedly inhibited Bruton’s tyrosine kinase enzyme activity and suppressed the B-cell receptor signaling pathway in Ramos cells. Moreover, it suppressed the expression of CD69, CD86, and MHC class II in mouse B lymphocytes and the production of TNF-α and MIP-1α in mouse macrophages and decreased bone resorption activity in mouse osteoclasts. Furthermore, it ameliorated the pathological changes in two rodent models of collagen-induced arthritis *in vivo*. TAS5315 improved bone mineral density and bone intensity. Thus, these results suggest that TAS5315 could be a promising therapeutic option for the treatment of rheumatoid arthritis.

## Introduction

Rheumatoid arthritis (RA) is an inflammatory autoimmune disease characterized by autoantibody production, synovial inflammation, and joint destruction. Its pathogenesis is triggered by various environmental factors and the personal genetic background of the patients. Individuals with a smoking history and double copies of the human leukocyte antigen-DR shared epitope (*HLA-DR SE*) gene exhibit greater positivity for anti-citrulline antibodies; thus, the risk of RA in these individuals is higher than that in nonsmokers without the *HLA-DR SE* gene [1, 2]. Moreover, RA is developed in a multistep process involving various effector cells. Recognition of self-antigens by T cells and B cells leads to the production of autoantibodies, followed by the deposition of immune complexes (ICs) in the synovium. The ICs bind to the Fcγ receptor (FcγR) on neutrophils and monocytes/macrophages; they further promote the production of prostaglandins, chemokines, and inflammatory cytokines, which leads to synovial inflammation [3]. Subsequently, cartilage destruction and bone erosion may occur in several joints of patients with RA. In the synovium, inflammatory cytokines such as tumor necrosis factor-α (TNF-α) and interleukin (IL)-1 promote the expansion of fibroblast-like synoviocyte (FLS) populations and expression of matrix metalloproteinases (MMPs) (particularly MMP-1, MMP-3, and MMP-13), which leads to cartilage destruction characterized by synovial hyperplasia and extracellular matrix (ECM) degradation [3, 4].

Bone erosion is a representative symptom in patients with RA and is triggered by excessive bone-resorbing activity of the osteoclasts [5]. Osteoclasts are differentiated from myeloid-derived progenitor cells by TNF-α, IL-6, macrophage colony-stimulating factor (M-CSF), and/or receptor activator for nuclear factor-κB (NF-κB) ligand (RANKL) [2]. RANKL is mainly produced by T cells, B cells, synovial fibroblast-like cells, osteoblasts, and osteocytes. It induces excess differentiation and activation of osteoclasts during inflammatory processes in RA [5]. In patients with RA, the activated osteoclasts exhibit high bone resorption activity, which leads to bone erosion via disruption of the balance between bone resorption and formation [6].

Numerous therapeutic agents have been approved for RA pathogenesis. Conventional synthetic disease-modifying anti-rheumatic drugs (csDMARDs), such as methotrexate, are used to treat RA; however, approximately 40% of patients newly diagnosed with RA fail to achieve low disease activity or disease remission with csDMARDs due to the lack of efficacy or poor tolerability. Furthermore, biologic DMARDs (targeting TNF-α, IL-6, cluster of differentiation (CD)80/86, CD20, etc.) are used to control disease progression in patients with active RA who fail treatment with csDMARDs or are intolerant of csDMARDs [7]. A new therapeutic class of targeted synthetic DMARDs (tofacitinib, baricitinib, etc.) that inhibit Janus kinases (JAKs) has been approved, and these drugs have gained immense attention as a new treatment option [8–10]. The options for RA pharmacotherapy have been increasing, as described previously; however, several unmet medical needs, such as insufficient effects against bone destruction and cartilage damage, remain due to either lack of efficacy or poor tolerability. New therapeutic drugs with novel mechanisms of action are required to address the unmet medical needs of patients with RA [5, 11, 12]. Several intracellular signaling molecules are reportedly involved in the pathogenesis of RA (e.g., PDGFR, nuclear factor κ-light-chain-enhancer of activated B cells (NF-κB), SYK, and Bruton’s tyrosine kinase (BTK)), and inhibition of these molecules could prove beneficial for patients with RA [3, 13].

BTK is a cytoplasmic non-receptor tyrosine kinase that belongs to the TEC family of kinases. It is broadly expressed in monocytes/macrophages, dendritic cells, mast cells, osteoclasts, and B cells (except for plasma cells) [14, 15]. In B cells, antigen recognition via the B-cell receptor (BCR) triggers intracellular signaling cascades that lead to BTK phosphorylation. Moreover, BTK induces phosphorylation of phospholipase C (PLC)γ2 and intracellular calcium mobilization and activates multiple transcription factors (NF-κB, nuclear factor of activated T-cells (NFAT), etc.), thereby promoting the survival, proliferation, and differentiation of B cells [16]. Current evidence suggests that targeting BTK could be a promising therapeutic option for autoimmune diseases such as RA due to the regulation of B-cell proliferation and function [17, 18]. In addition, BTK is a key molecule in BCR signaling, as well as in FcγR and RANK signaling, and it regulates the function of various effector cells associated with the pathogenesis of RA [15, 19, 20]. Various BTK inhibitors have demonstrated anti-inflammatory effects by inhibiting the functions of immune cells in preclinical experimental models; these inhibitors are under clinical development for the treatment of autoimmune diseases [21–28].

In the present study, we characterized TAS5315, a highly potent and selective BTK inhibitor, to evaluate its utility as a potential therapeutic drug for the treatment of RA by assessing its inhibitory effects on the functions of various effector cells associated with RA progression. TAS5315 exhibited therapeutic effects against synovial inflammation, pannus formation, cartilage destruction, and bone erosion in rodent models of RA. In particular, TAS5315 improved bone mineral density (BMD) and bone strength. These findings suggest that TAS5315 could be a potential therapeutic candidate for RA as it inhibits the functions of effector cells associated with RA pathogenesis.

## Materials and methods

### Materials

The following antibodies were used: rabbit anti-human phosphorylated BTK (tyrosine 223) monoclonal antibody (EP420Y) (ab68217), mouse anti-human p84 monoclonal antibody (ab487) (Abcam, Cambridge, UK), mouse anti-human BTK monoclonal antibody (611117), fluorescein isothiocyanate-conjugated rat anti-mouse CD45R/B220 monoclonal antibody (553088), phycoerythrin-cyanine 7-conjugated anti-mouse CD45R/B220 monoclonal antibody (552772), allophycocyanin-conjugated rat anti-mouse CD86 monoclonal antibody (558703), brilliant violet 421-conjugated rat anti-mouse I-A/I-E monoclonal antibody (562564) (BD Biosciences, San Jose, CA, USA), APC-conjugated Armenian hamster anti-mouse CD69 monoclonal antibody (17-0691-82; Thermo Fisher Scientific, Waltham, MA, USA), rabbit anti-human G3PDH/GAPDH polyclonal antibody (2275-PC-100; Trevigen, Gaithersburg, MD, USA), rabbit anti-mouse BTK monoclonal antibody (8547), rabbit anti-human phosphorylated PLCγ2 (tyrosine 1217) polyclonal antibody (3871), rabbit anti-human PLCγ2 polyclonal antibody (3872), rabbit anti-human phosphorylated AKT (serine 473) monoclonal antibody (4075), rabbit anti-human AKT polyclonal antibody (9272), rabbit anti-human phosphorylated ERK (threonine 185 and tyrosine 187) monoclonal antibody (4370), rabbit anti-human ERK polyclonal antibody (9102) (Cell Signaling Technology, Danvers, MA, USA), mouse anti-human NFATc1 monoclonal antibody (MABS409), and mouse anti-bovine β-tubulin monoclonal antibody (05–661) (Merck Millipore, Darmstadt, Germany).

TAS5315 was synthesized by Taiho Pharmaceutical Co., Ltd. (Tokyo, Japan) using a previously described method (publication no. WO2015022926A1, published in 2015).

### Cell culture

All cells were cultured in an incubator at 37°C and 5% CO_2_. Ramos human B cell lymphoma (CRL-1596) (American Type Culture Collection; ATCC, Manassas, VA, USA) was maintained in RPMI-1640 (Wako Pure Chemical Industries, Osaka, Japan) containing 10% fetal bovine serum (FBS; Thermo Fisher Scientific) and 1% penicillin-streptomycin (Pen-Strep; Thermo Fisher Scientific). Ramos-Blue cells (rms-sp, InvivoGen, San Diego, CA, USA) were maintained in IMDM (Thermo Fisher Scientific) containing 10% FBS, 1% Pen-Strep, and 100 μg/mL of Normocin and Zeocin (InvivoGen). The cell lines were obtained directly from each company.

### Experimental animals

The animals used in the present study were DBA/1JnCrlj mice, C57BL/6J mice, and LEW/CrlCrlj rats from Charles River Laboratories Japan (Yokohama, Japan). For administration, TAS5315 and prednisolone were dissolved in a solution of 0.5% hydroxypropyl methylcellulose (Shinetsu Chemical, Tokyo, Japan).

All animal experiments were approved by the Institutional Animal Experiments Ethics Committee of Taiho Pharmaceutical Co., Ltd. and were performed in compliance with the guidelines established by the aforementioned committee. All surgeries, including sacrifice, were performed under isoflurane inhalation to minimize suffering. Experimental animals upon conclusion of the experiment and sampling animals were sacrificed immediately by dislocation of the cervical vertebra (mice) or exsanguination (rats).

### Biochemical assays

A mobility shift assay (PerkinElmer, Waltham, MA, USA) was performed to measure the kinase inhibitory activity. ProfilerPro Kinase Selectivity Assay (PerkinElmer) was used to determine the half-maximal inhibitory concentration (IC_50_) against eight kinases (BTK, BMX, BLK, EGFR, ITK, JAK3, TEC, and TXK) according to the manufacturer’s instructions. Various concentrations of TAS5315 or dimethyl sulfoxide (DMSO) were added to each well of the enzyme plate, which was further incubated for 15 min. Thereafter, the substrate solutions were transferred from the substrate plate to the enzyme plate, and the enzyme plate was incubated for 90 min. A termination buffer was added to the plate, and the plates were read using LabChip EZ Reader II (PerkinElmer).

### BCR signaling in Ramos cells

To examine phosphorylation status of kinases on BCR signaling pathway, Ramos cells (2×10^6^ cells/well) were treated with various concentrations of TAS5315 or DMSO for 1 h in FBS-free conditions, and then stimulated with goat F(ab′)_2_ anti-human immunoglobulin (Ig)M LE/AF (5 μg/mL; SouthernBiotech, Birmingham, AL) for 10 min. Protein extracts were prepared from the cells and were subjected to sodium dodecyl sulfate-polyacrylamide gel electrophoresis (SDS-PAGE) and western blotting to assess the expression of phosphorylated proteins and total proteins.

To examine calcium flux, Ramos cells (1×10^6^ cells/well) were treated with various concentrations of TAS5315 or DMSO for 30 min under FBS-free conditions before anti-IgM stimulation (5 μg/mL). Intracellular calcium levels were determined using a FLIPR Calcium 5 Assay Kit (Molecular Devices, Sunnyvale, CA, USA), as previously reported [29].

Ramos-Blue cells stably express an NF-κB/activator protein-1 (AP-1)-inducible secreted embryonic alkaline phosphatase (SEAP) reporter gene [30]. These cells (2×10^5^ cells/well) were treated with various concentrations of TAS5315 or DMSO for 30 min under FBS-free conditions and then incubated with anti-IgM (5 μg/mL) for 24 h. NF-κB/AP-1 activation (SEAP expression / optical density (OD) value) was assessed as previously reported [31].

### CD69, CD86, and major histocompatibility complex (MHC) class II expression on mouse B cells

Mouse splenocytes were isolated from DBA/1JnCrlj mice and seeded in RPMI-1640 medium containing 10% FBS and 1% Pen-Strep. Thereafter, the cells (5×10^5^ cells/well) were incubated with goat F(ab′)_2_ anti-mouse IgM-UNLB (5 μg/mL; SouthernBiotech) and various concentrations of TAS5315 or DMSO for 24 h and further stained with fluorescent-labeled antibodies against B220, CD69, CD86, and MHC class II (I-A/I-E). The mean fluorescence intensities corresponding to CD69, CD86, and MHC class II on B220^+^ lymphocytes (S1 Fig) were assessed using a FACSVerse flow cytometer (BD Biosciences).

### RANKL signaling in bone marrow-derived macrophages (BMDMs)

In these experiments, bone marrow cells were prepared from the femora and tibiae of DBA/1JNCrlj or C57BL/6J mice.

To examine BTK phosphorylation, BMDMs were differentiated from bone marrow cells via treatment with M-CSF (20 ng/mL) for 7 days and were then cultured at 4×10^5^ cells/well in the presence of M-CSF (20 ng/mL) and RANKL (100 ng/mL) with various concentrations of TAS5315 or DMSO for 24 h. After 24 h of stimulation with M-CSF and RANKL, protein extracts were prepared from the cells and subjected to SDS-PAGE and western blotting to assess protein phosphorylation and the expression of total proteins.

To examine the nuclear translocation of NFATc1, BMDMs (3.75×10^6^ cells/dish) were differentiated from bone marrow cells via a 48-h treatment with M-CSF (10 ng/mL) and were then stimulated with M-CSF (10 ng/mL) and RANKL (100 ng/mL) in the presence of DMSO or TAS5315 for 24 h. After 24 h of stimulation with M-CSF and RANKL, nuclear and cytosolic extracts of BMDMs were prepared using an NE-PER nuclear extraction kit (Thermo Fisher Scientific). SDS-PAGE and western blotting were performed to assess the expression of total proteins.

### Osteoclast differentiation

Osteoclast differentiation assays were performed using the OCP BulletKit (Lonza Japan, Tokyo, Japan). Human osteoclast precursor cells (5×10^3^ cells/well) were cultured in the presence of M-CSF (33 ng/mL) and RANKL (66 ng/mL), with various concentrations of TAS5315 or DMSO. After incubation for 4 days, the cells were fixed with 10% neutral-buffered formalin and stained with using a tartrate-resistant acid phosphatase (TRAP) staining kit (Cosmo Bio, Tokyo, Japan) and DAKO REAL Hematoxylin (Dako, Glosturp, Denmark). The number of TRAP-positive multinucleated cells (>3 nuclei per cell, >50 μm in width) was considered as the mature osteoclast count.

### Bone resorption by human osteoclasts

Human osteoclast precursor cells (Lonza Japan) were seeded at 1.5×10^4^ cells/well on an OsteoAssay Human Bone Plate (Lonza, Japan). The cells were cultured in the presence of M-CSF (33 ng/mL) and RANKL (66 ng/mL) with various concentrations of TAS5315 or DMSO, according to the manufacturer’s instructions, and the medium was exchanged on days 4, 7, and 11. The level of C-terminal telopeptides of type I collagen (CTX-I) in the culture supernatant on day 14 was determined via CTX-I enzyme-linked immunosorbent assay (ELISA) (Immunodiagnostic Systems, Boldon, UK).

### Pit formation by mouse osteoclasts

Mouse osteoclast precursor cells (Cosmo Bio, Tokyo, Japan) were seeded at 8×10^4^ cells/well on dentin slices (Dentin Slice, from Ivory, Thin Type; Wako Pure Chemical Industries) and incubated with various concentrations of TAS5315 or DMSO for 16 days according to the manufacturer’s instructions. The medium was exchanged on days 4, 7, 11, and 14. On day 16, the dentin slices were washed with 1 M ammonia and stained with hematoxylin. The resorbed area on dentin slices was analyzed using the Image Pro version 7.0 software (MediaCybernetics, Rockville, MD, USA).

### BTK occupancy assay with rat peripheral blood mononuclear cells (PBMCs)

TAS-6565, a chemical probe comprising boron-dipyrromethene (BODIPY) FL fluorophore-conjugated TAS5315, binds to BTK. PBMCs were isolated via density gradient centrifugation (Ficoll-Paque PREMIUM 1.084; GE Healthcare, Buckinghamshire, UK) from the peripheral blood of rats 6 h after TAS5315 administration. Cells were lysed, and the extracted protein (2 mg/mL) was incubated with TAS-6565 (1 μM) for 30 min at 37°C. Thereafter, proteins from each sample were resolved via SDS-PAGE, and fluorescence derived from the BODIPY FL fluorophore was detected using a Typhoon Trio^+^ (Ex/Em = 488/520 nm; GE Healthcare). Eventually, the gel was blotted onto a membrane, and whole BTK expression levels were detected via western blotting.

### Rat and mouse collagen-induced arthritis (CIA) models

For the rat CIA model, experimental arthritis was induced and volumes of both hind paws were measured as previously described [29]. TAS5315 or prednisolone (1 mg/kg/day) was administered orally for 21 consecutive days from the day of the second immunization.

For the mouse CIA model, male DBA/1JNCrlj mice (7 weeks old) were injected with complete Freund’s adjuvant (CFA) (BD Biosciences) containing 2 mg/mL of bovine type II collagen at a site on the back (each 0.1 mL) and were boosted 21 days later in a similar manner. Arthritis scores were determined as previously reported [29]. For histopathological examinations, TAS5315 or prednisolone (3 mg/kg/day) was administered orally for 15 consecutive days from 7th day after the second immunization. For micro-computed tomography (CT) examinations, TAS5315 or prednisolone (3 mg/kg/day) was administered orally for 21 consecutive days from the 11th day after the second immunization.

### Histopathological evaluation in the mouse CIA model

On the day after the final administration, the four limbs of each mouse were harvested and decalcified in 10% formalin. The specimens were then embedded in paraffin blocks, cut into sections, stained with hematoxylin and eosin, and randomly selected for microscopic examination. Histopathological assessments were performed as described previously [29]. This experiment was carried out in the laboratory of the BoZo Research Center Inc. (Ibaraki, Japan).

### Micro-CT imaging in the CIA model

The bone morphology of CIA mice was evaluated via micro-CT using Cosmo Scan (Rigaku, Tokyo, Japan). Hind paws of the mice were scanned using the following scanning conditions: 90 kV; 88 μA; field of view, 25 mm; 2 min. The BMD was calculated using the Analyze version 11.0 software (Biomedical Imaging Resource, Mayo Clinic, Rochester, MN). The bone was determined to have a BMD value ≥480 mg/cm^3^, and the region of interest for calculating the BMD included the talus and cuboid. BMD was calibrated using a QRM-MicroCT-HA phantom (Quality Assurance in Radiology and Medicine, Moehrendorg, Germany) that was scanned on each day when micro-CT of animals was performed. Calibration was performed via scanning phantoms with densities of 0, 50, 200, 800, and 1200 mg HA/cm^3^, and data analysis was performed using the Analyze11.0 Bone Mineral Density Analysis procedure.

### Statistical analysis

Results are reported as mean ± standard deviation (SD) for *in vitro* experiments or mean ± standard error of the mean (SEM) for *in vivo* experiments. Statistical analyses and determination of IC_50_ values were performed using EXSUS version 8.0.0 software (CAC Exicare, Tokyo, Japan). Statistical significance was assessed using the Dunnett test or Steel test for more than three groups or the Student’s *t*-test or Wilcoxon test for two groups. In all analyses, statistical significance was set at *P* < 0.05.

## Results

### Potency and selectivity of TAS5315 in the enzymatic assay

TAS5315 potently inhibited the kinase activity of human recombinant BTK with an IC_50_ of 2.0 nM (Table 1). We performed an enzymatic assay for 186 kinases to determine the potency and selectivity of TAS5315. TAS5315 inhibited 7 of the 186 kinases (BLK, TEC, TXK, ITK, EGFR, JAK3, and HER4) with >50% inhibition at 100 nM (S2 Fig). Thereafter, we calculated the IC_50_ of TAS5315 for these seven kinases and BMX, which is a member of the TEC family of kinases. Among the tested kinases, TAS5315 exhibited high selectivity against TEC family kinases (TEC, BMX, ITK, and TXK) and BLK (Table 1).

**Table 1:** Potency and selectivity of TAS5315 in enzymatic assays.

Kinase name	IC_50_ (nM)	selectivity
BTK	2.0	-
TEC	0.55	×0.28
BMX	0.85	×0.43
BLK	1.6	×0.80
ITK	6.2	×3.1
TXK	6.5	×3.8
EGFR	21	×10.5
JAK3	60	×30
HER4	71	×36

### Inhibitory effects of the BCR signaling pathway by TAS5315

**Fig 1:**
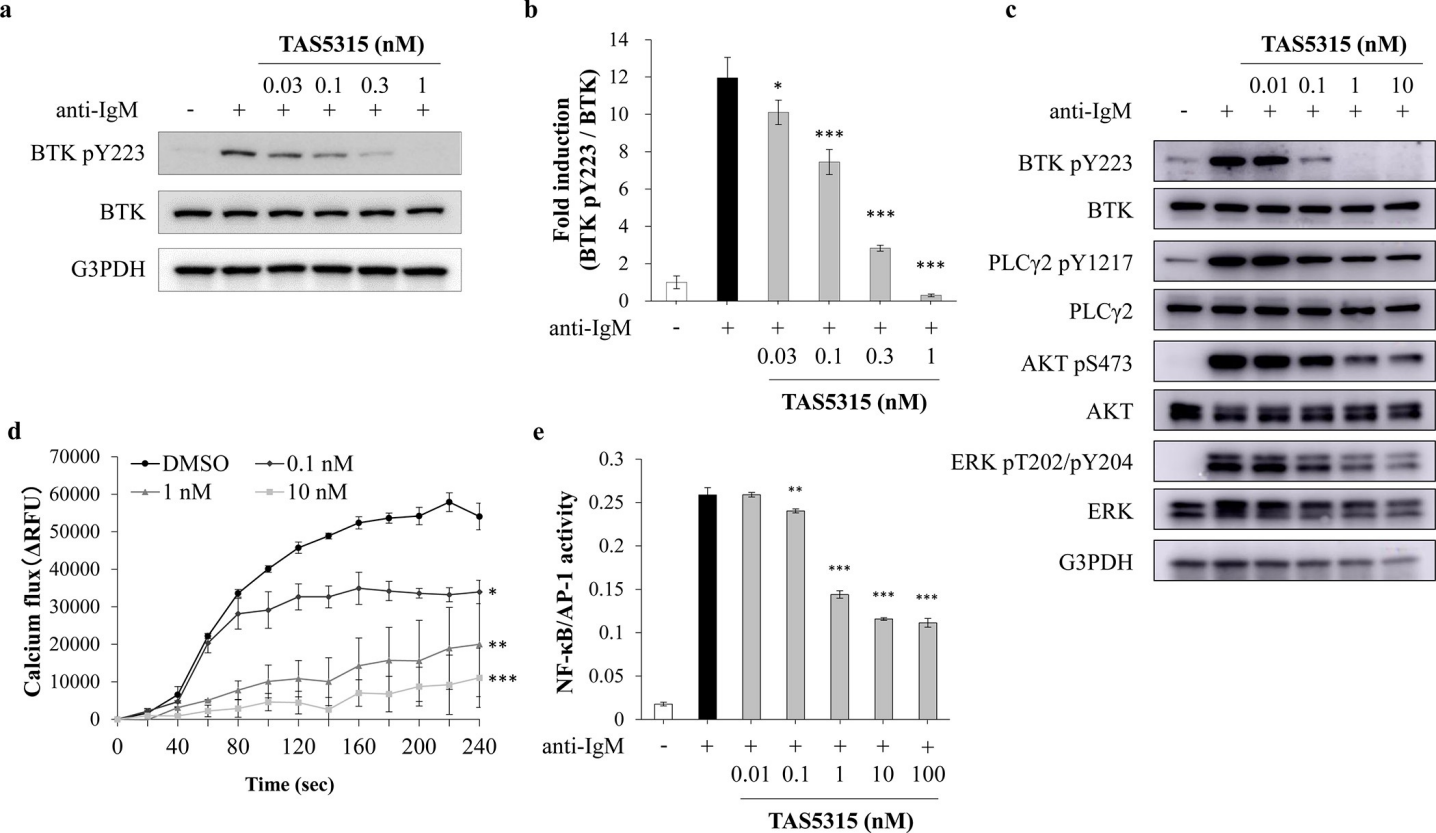
TAS5315 inhibits the phosphorylation of BTK and its downstream signaling pathway in Ramos cells. (a, c) Phosphorylation of various kinases of the BCR signaling pathway induced by anti-IgM stimulation (5 μg/mL) for 10 min in Ramos cells. TAS5315 was applied for 1 h as pretreatment. Immunoblots provide representative data from three independent experiments. (b) Densitometric analysis of protein amounts to determine the relative levels of phosphorylated BTK compared to that of total BTK. (d) Intracellular calcium flux in Ramos cells induced by anti-IgM stimulation (5 μg/mL) at the indicated times. TAS5315 was treated for 30 min before anti-IgM stimulation. (e) NF-κB and AP-1 transcriptional activity in Ramos-Blue cells stimulated with anti-IgM (5 μg/mL) for 24 h. TAS5315 was treated for 30 min before anti-IgM stimulation. SEAP level (OD value) in the culture supernatant was measured as an index of NF-κB and AP-1 transcriptional activity. All experiments were performed in triplicate, and the numerical data are presented as mean ± SD. *P<0.05, **P<0.01, ***P<0.001 compared with the DMSO group (Dunnett test for TAS5315 groups).

BTK is associated with the proliferation, survival, and differentiation of B cells by regulating the BCR signaling pathway [16]. During the pathogenesis of RA, abnormally activated B cells produce autoantibodies and cytokines and present autoantigens [3]. Therefore, we examined the effects of TAS5315 on the BCR signaling pathway activated by anti-IgM stimulation in Ramos cells, a B-cell line derived from Burkitt’s lymphoma. Moreover, TAS5315 dose-dependently inhibited the phosphorylation of BTK with an IC_50_ value of 0.15 nM in Ramos cells stimulated with anti-IgM (Fig 1A and 1B). Additionally, it inhibited the phosphorylation of PLCγ2, the substrate of BTK, and the phosphorylation of further downstream molecules such as AKT and ERK (Fig 1C). Phosphorylated PLCγ2 generates inositol-1, 4, 5-trisphosphate and initiates the mobilization of intracellular calcium from the endoplasmic reticulum [16, 32]. Furthermore, TAS5315 decreased intracellular calcium mobilization by stimulation with anti-IgM in Ramos cells (Fig 1D). Enhanced BCR signaling induces the activation of various transcription factors (NF-κB, NFAT, and AP-1) and regulates the expression of downstream responsive genes by these transcription factors [16, 33]. To assess the effects of TAS5315 on the activation of NF-κB and AP-1 in BCR signaling, we used Ramos-Blue cells that stably expressed an NF-κB/AP-1-inducible SEAP reporter gene. Ramos-Blue cells can indicate NF-κB and/or AP-1 transcriptional activation by releasing SEAP into the culture medium [30]. TAS5315 dose-dependently suppressed NF-κB/AP-1 transcriptional activity in Ramos-Blue cells stimulated with anti-IgM (Fig 1E).

**Fig 2:**
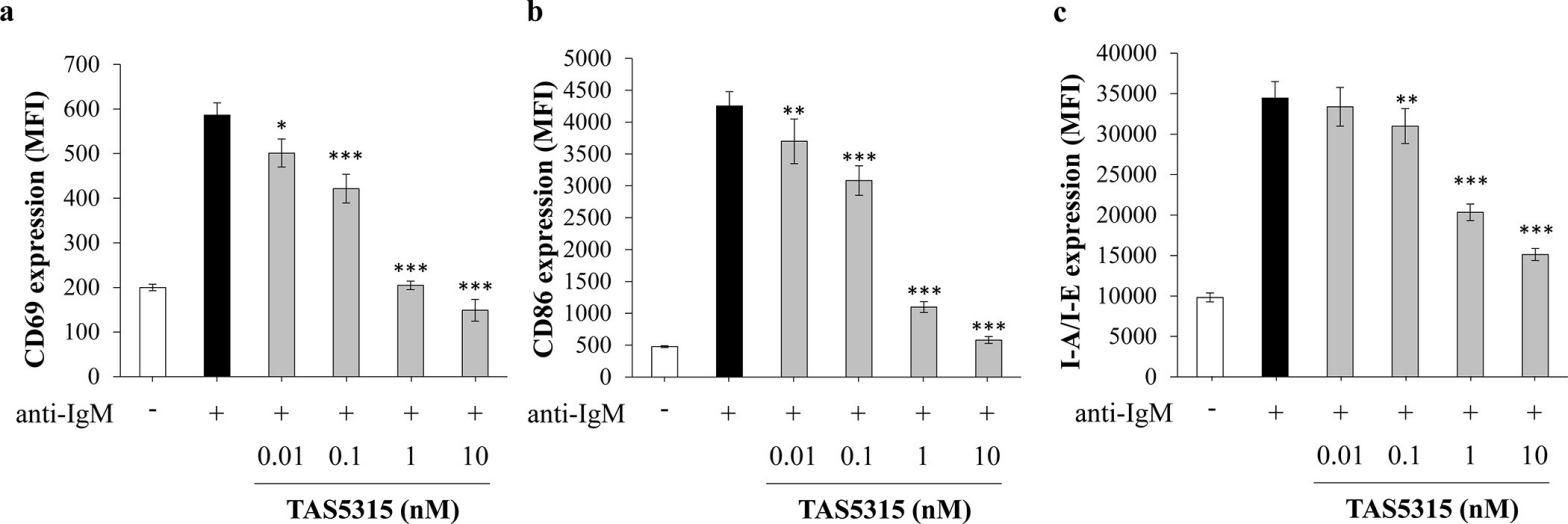
TAS5315 inhibits cell surface expressions of CD69, CD86, and MHC class II on mouse splenic B cells. (a–c) Cell surface expressions of CD69, CD86, and I-A/I-E on mouse B220^+^ B lymphocytes stimulated with anti-IgM (5 μg/mL). TAS5315 treatment was performed simultaneously with anti-IgM stimulation. All experiments were performed in triplicates, and the numerical data are presented as mean ± SD. *P<0.05, **P<0.01, ***P<0.001 compared with the DMSO group (Dunnett test for TAS5315 groups).

Consistent with these suppressive effects of TAS5315 on the BCR signaling pathway, TAS5315 also suppressed the surface expression of the lymphocyte activation marker CD69 and T-cell stimulatory molecule CD86 and MHC class II (I-A/I-E) on mouse splenic B cells stimulated with anti-IgM (Fig 2A–2C).

The BioMAP panel was used to evaluate the efficacy and selectivity of TAS5315 for inhibiting immune-related signaling pathways. The BioMAP panel is a research tool used to identify the mechanism of action of a target compound and predict any potential adverse effects with 12 primary human monoculture or co-culture systems, such as the BT system, which comprises a co-culture of B cells and PBMCs under stimulation with anti-IgM and T-cell receptor ligands [34–36]. In the present study, this panel was used to evaluate the efficacy and selectivity of TAS5315 in inhibiting immune-related signaling pathways. When TAS5315 was applied at concentrations of 4.6, 14, 41, and 120 nM in this panel, it markedly inhibited the production of secreted IgG and various cytokines (soluble IL-2, IL-17A, and TNF-α) in the BT system over log ratio values of -0.5, without exhibiting major changes in various readout parameters in other systems (S3 Fig). Consistent with the results from enzyme assays (Table 1 and S2 Fig), these data support that TAS5315 is a potent and selective BTK inhibitor.

### Suppressive effects of TAS5315 on the monocyte/macrophage lineage

BTK regulates BCR signaling as well as FcγR and RANK-related signaling pathways in the effector cells associated with the pathogenesis of RA [15, 19]. After FcγR recognizes ICs formed from autoantibodies in macrophages, IC-activated macrophages start producing inflammatory cytokines and chemokines in autoimmune diseases such as RA [37–39]. We examined the effects of TAS5315 on the inflammatory response in THP-1 cells and mouse BMDMs by cross-linking FcγR with IgG during the treatment of TAS5315. TAS5315 dose-dependently suppressed the production of TNF-α in THP-1 cells and that of TNF-α and MIP-1α in mouse BMDMs (S4A–S4C Fig).

In RA-associated cartilage destruction, FLSs play an important role in synovial hyperplasia and ECM degradation. FLSs are activated by inflammatory cytokines such as TNF-α, which induces MMP3 production, and, thus, ECM degradation [4]. Here, TAS5315 did not directly inhibit FLS proliferation or MMP-3 expression by FLSs under TNF-α stimulation (S4D and S4E Fig). However, the FLS proliferation and MMP-3 production in FLSs were induced by exposure to the culture supernatant of IgG-activated THP-1 cells and were inhibited by treating THP-1 cells with TAS5315 (S4F and S4G Fig).

### Suppressive effects of TAS5315 on RANKL signaling and osteoclasts

Bone metabolism is a complex process of bone turnover, which includes bone resorption by osteoclasts and bone formation by osteoblasts. In patients with RA, synovial inflammation by inflammatory cells in the pannus leads to abnormal bone erosion by osteoblasts [40]. BTK is reportedly associated with RANKL signaling [15], and some BTK inhibitors exhibit inhibitory effects on osteoclast function [25, 26, 41]. We also evaluated the effects of TAS5315 on RANKL signaling and osteoclast function.

**Fig 3:**
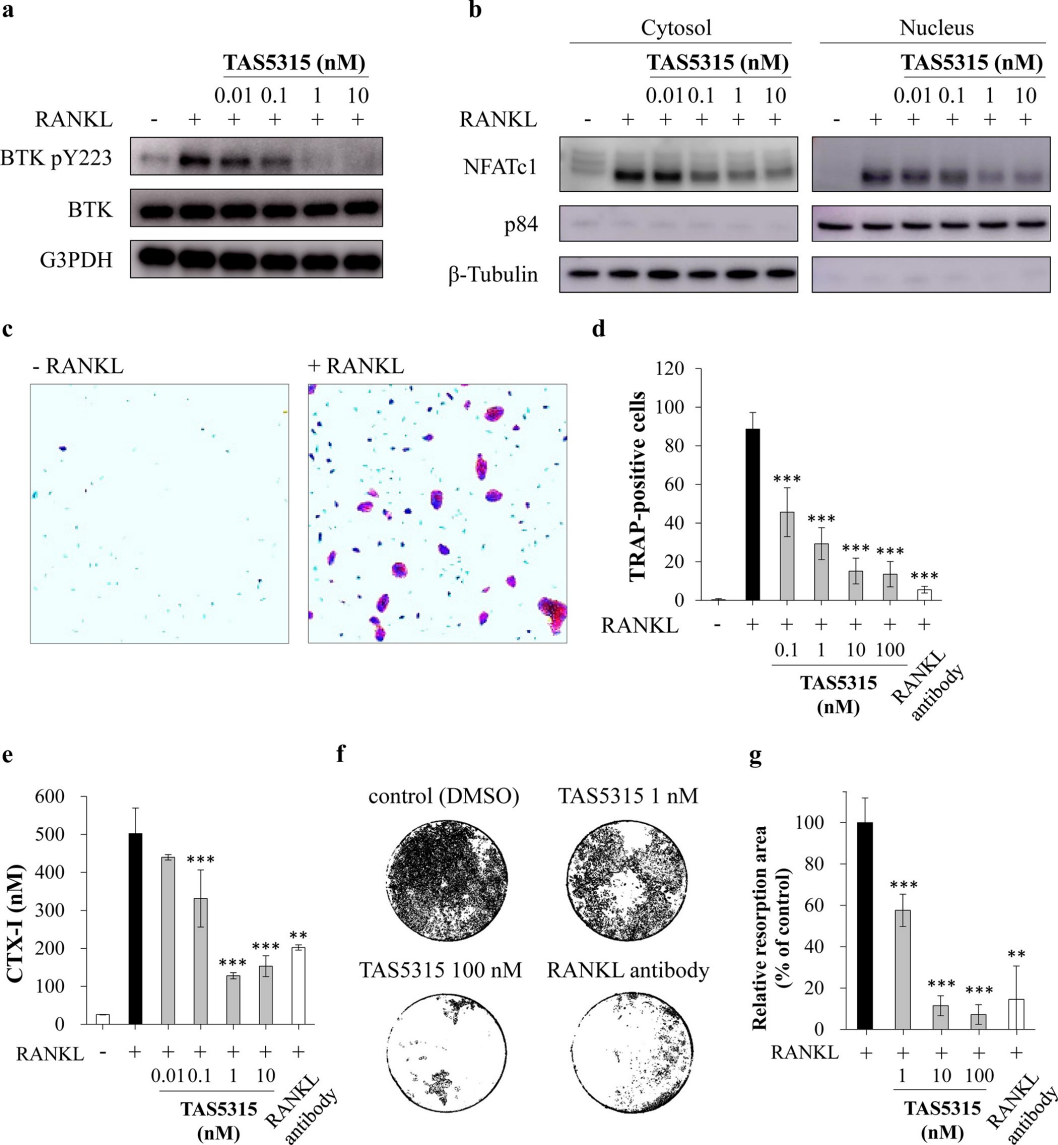
Effect of TAS5315 on macrophages and osteoclasts. (a) Phosphorylation of BTK induced by RANKL stimulation (100 ng/mL) for 24 h in M-CSF-induced murine macrophages. Macrophages were simultaneously subjected to TAS5315 and RANKL stimulation. (b) Nuclear translocation of NFATc1 induced by RANKL stimulation (100 ng/mL) for 24 h in M-CSF-induced murine macrophages. M-CSF-induced murine macrophages were simultaneously subjected to TAS5315 and RANKL stimulation. The figures of immunoblot experiments (a, b) represent the data of three independent experiments. (c, d) Human osteoclast differentiation was induced by RANKL (66 ng/mL) and M-CSF (33 ng/mL) in the presence of DMSO, TAS5315, or anti-RANKL antibody (0.1 μg/mL) for 4 days. (c) Representative images of TRAP-positive osteoclasts. (d) TRAP-positive cells with 3 or more nuclei (cell size ≥50 μm) were counted as osteoclasts. (e) CTX-I levels in the supernatant of human osteoclasts treated with M-CSF (33 ng/mL) and RANKL (66 ng/mL) in the presence of DMSO, TAS5315, or anti-RANKL antibody (0.1 μg/mL) for 14 days. (f, g) Pit formation by mouse osteoclasts treated with RANKL (25 ng/mL) and M-CSF (50 ng/mL) in the presence of DMSO, TAS5315, or anti-RANKL antibody (0.1 μg/mL) for 16 days. (f) Representative images of pit formation by mouse osteoclast (DMSO, TAS5315 100 nM, anti-RANKL antibody). (g) Quantitative analysis of the relative pit formation area by mouse osteoclasts. Experiments (c–g) were performed in triplicates and the numerical data (d, f, g) are presented as mean ± SD. *P<0.05, **P<0.01, ***P<0.001 compared with DMSO group (Dunnett test for TAS5315 groups or Student’s t-test for anti-RANKL antibody).

First, we used mouse BMDMs to evaluate the effects of TAS5315 on BTK phosphorylation and nuclear localization of NFAT by RANKL stimulation. In BMDMs stimulated with RANKL, TAS5315 dose-dependently inhibited the phosphorylation of BTK and translocation of NFAT (Fig 3A and 3B). Osteoclast differentiation from precursor cells was induced via stimulation with RANKL and M-CSF for 4 days and was evaluated by enumerating the TRAP-positive cells (Fig 3C). TAS5315 and the anti-RANKL antibody decreased the number of TRAP-positive cells (Fig 3D and S5 Fig). Next, we cultured osteoclast precursor cells with RANKL and M-CSF for 14 days and evaluated the effects of TAS5315 on bone resorption activity. TAS5315 suppressed both the production of CTX-I, bone resorption marker in human osteoclasts, and bone resorption activity in mouse osteoclasts (Fig 3E–3G and S5 Fig).

### Therapeutic efficacy of TAS5315 against inflammation in rodent CIA models

**Fig 4:**
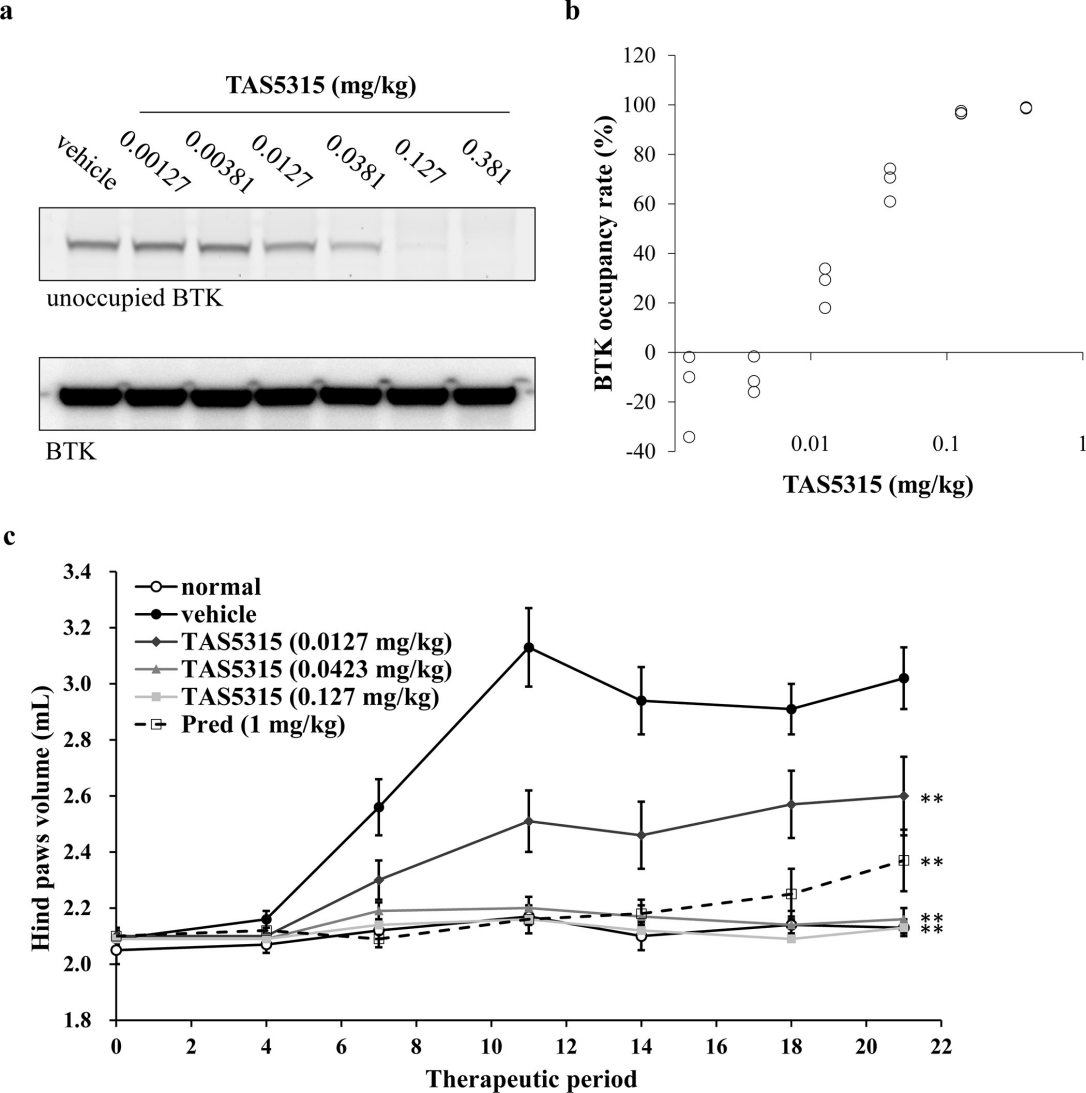
Anti-inflammatory effect of TAS5315 in a rat CIA model. (a, b) BTK occupancy by TAS5315 in PBMCs from rats at 6 h after administration. BTK that was not occupied by TAS5315 was detected with a BODIPY probe (*n* = 3). (a) Representative data for the BTK occupancy assay. (b) Each BTK occupancy rate indicates the percentage (%) at which the fluorescence intensity of background and vehicle-treated rat PBMC samples were 0% and 100%, respectively. (c) Hind paw volumes in the rat CIA model. Experimental arthritis was induced by two immunizations with collagen and incomplete Freund’s adjuvant. For 21 days from the second immunization (Day 1), rats were administered once daily with vehicle, TAS5315, or prednisolone (Pred) (*n* = 6–10 per group). Data are presented as mean ± SEM. ** *P*<0.01 compared with the vehicle group (Dunnett test for TAS5315 groups, Student’s t-test for Pred group).

The results of cell-based assays indicated that TAS5315 suppresses the functions of effector cells associated with disease progression in RA. We investigated whether TAS5315 can improve the symptoms of RA in several experimental arthritis models. Moreover, we performed a pharmacokinetic (PK)-pharmacodynamic (PD) study using a BTK occupancy assay to evaluate BTK inhibition by TAS5315 in rats. PK parameters (AUC_0-24h_, C_max_, and T_max_) were determined from the measured TAS5315 concentrations in rat plasma after the oral administration of TAS5315. The plasma concentrations of TAS5315 increased in a dose-dependent manner over a dose range of 0.00381–0.381 mg/kg (S1 Table). The PD study was performed by quantifying the BTK occupancy of TAS5315 in rat PBMCs after oral administration of TAS5315 using BODIPY-conjugated TAS5315 (TAS-6565). Furthermore, the BTK occupancy rate of TAS5315 increased in a dose-dependent manner, and TAS5315 revealed full occupancy of BTK at doses ≥0.127 mg/kg in PBMCs isolated from rats after administering TAS5315 (Fig 4A and 4B). Next, we evaluated the effect of TAS5315 on joint swelling in a rat model of CIA. When TAS5315 was administered orally for 21 days starting from the day of the second immunization, TAS5315 significantly and dose-dependently suppressed the swelling of the hind paws in CIA rats compared with that in vehicle-treated rats (Fig 4C).

**Fig 5:**
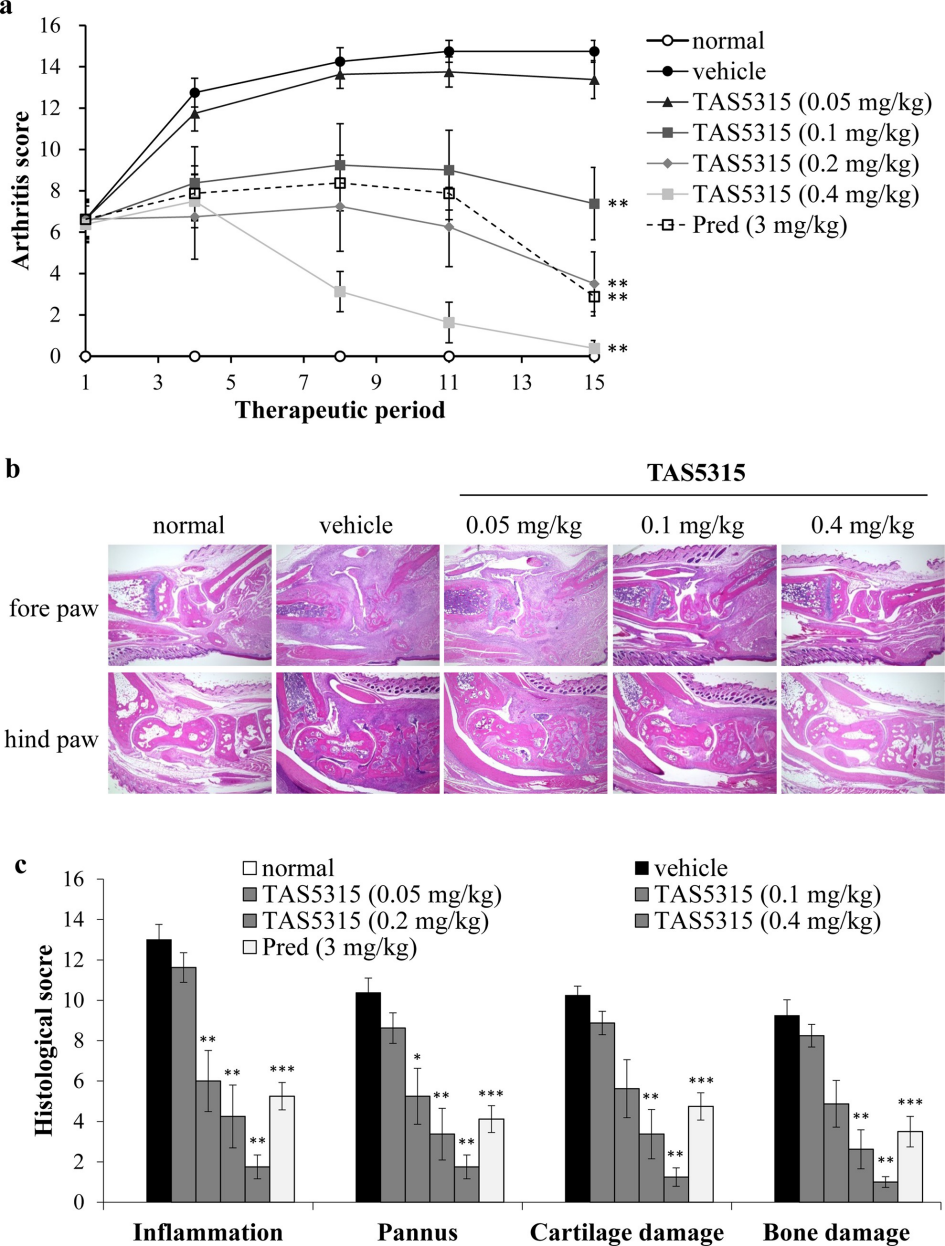
Therapeutic effects of TAS5315 in a mouse CIA model. (a) Arthritis score in the mouse CIA model. Experimental arthritis was induced by two immunizations with collagen and CFA. Mice were administered vehicle, TAS5315, or prednisolone (Pred) once daily for 15 days from 7th day after the second immunization (day 1). (b, c) Histological changes in the normal mice and CIA mice on the day following final administration. (b) Representative photomicrographs of hematoxylin-eosin staining (original magnification, 4×). (c) Histopathology scores from the forepaws and hind paws (both sides) were evaluated based on inflammation, pannus, cartilage damage, and bone damage. Data are presented as mean ± SEM. *P<0.05, **P<0.01 compared with the vehicle group (Steel test for TAS5315 groups, Wilcoxon test for Prednisolone group, *n* = 6–8 per group).

Next, we examined whether TAS5315 exhibited a therapeutic effect using an established mouse model of CIA. TAS5315 was administered orally at four doses (0.05, 0.1, 0.2, and 0.4 mg/kg) for 15 days. Consistent with results from the rat CIA model, TAS5315 decreased arthritis scores in a dose-dependent manner and exhibited significant therapeutic effects on disease progression at a dose of 0.1 mg/kg/day (Fig 5A). Fig 5B illustrates representative histopathological images of normal mice, vehicle-treated mice, and TAS5315 (0.05, 0.1, or 0.4 mg/kg)-treated CIA mice on day 15. Vehicle-treated mice exhibited infiltration of inflammatory cells into the synovium and formation of pannus in the fore- and hind paws. In contrast, partial improvement of disease severity in the fore- and hind paws was observed in CIA mice treated with 0.1 mg/kg TAS5315. The decrease in these pathological changes was the greatest in CIA mice treated with 0.4 mg/kg TAS5315; moreover, their paws were similar to those of normal mice. TAS5315 decreased the histopathological scores (inflammation, pannus, cartilage damage, and bone erosion) in a dose-dependent manner, and these scores in TAS5315-treated CIA mice were markedly lower (0.1 mg/kg) than those in the vehicle-treated mice (Fig 5C).

As TAS5315 ameliorated the arthritis score in an established mouse CIA model, we further measured whether TAS5315 reduced the levels of TNF-α, IL-1β, and IL-6 in synovial fluid exudates derived from the hind paws. These inflammatory factors are associated with inflammation, cartilage destruction, and bone damage in RA pathogenesis [42]. Consistent with the decreases in arthritis score by TAS5315 (S6A Fig), TAS5315 markedly decreased the levels of TNF-α, IL-1β, and IL-6 (S6B Fig).

### Therapeutic efficacy of TAS5315 against bone damage in rodent CIA models

**Fig 6:**
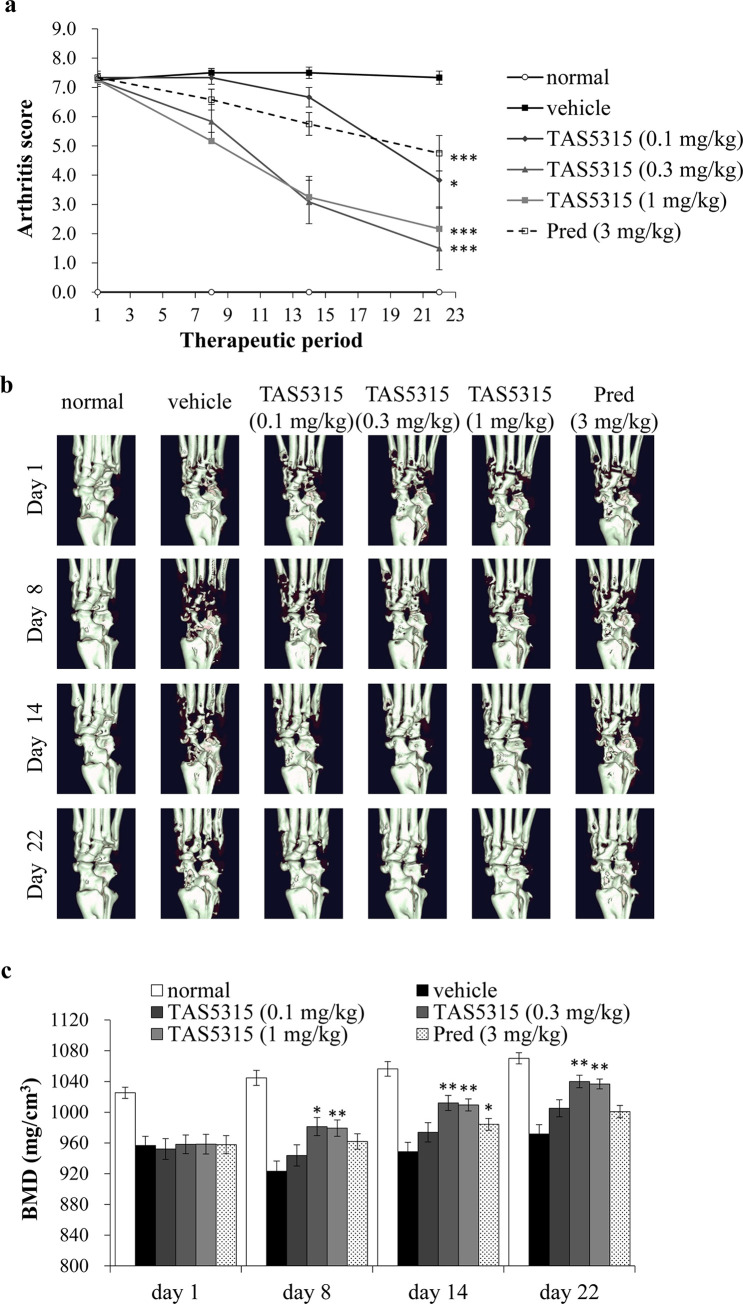
Therapeutic effect of TAS5315 on bone destruction in a mouse CIA model. (a) Arthritis score of hind paws in a mouse CIA model. Experimental arthritis was induced by two immunizations with collagen and CFA. Mice were administered vehicle, TAS5315, or prednisolone (Pred) once daily for 21 days from the 11th day after the second immunization (day 1). (b) Representative images of the right hind paw obtained by micro-CT on days 1, 8, 14, and 22. (c) The vertical axis depicts BMD values as determined by micro-CT analysis of both hind paws. Data are presented as mean ± SEM (*n* = 6–12 per group). *P<0.05, **P<0.01, ***P<0.001 compared with the vehicle group (Steel test for TAS5315 groups, Wilcoxon test for Pred group).

TAS5315 suppressed osteoclast differentiation and bone resorption activity *in vitro*, as illustrated in Fig 3. We evaluated the effects of TAS5315 on bone destruction in an established CIA mouse model. Experimental arthritis was induced via two immunizations using collagen and CFA. Mice were administered vehicle, TAS5315, or prednisolone once daily for 21 days from day 1 (11 days after the second immunization), when arthritis was established and bone destruction was observed in the mouse CIA model. TAS5315 and prednisolone markedly decreased arthritis scores in the CIA model compared with those in vehicle-treated mice; moreover, TAS5315 dose-dependently decreased arthritis scores at a dose of 0.1 mg/kg (Fig 6A). Fig 6B illustrates the representative micro-CT images of bone in the hind paws of the mouse CIA model; furthermore, TAS5315 (0.3 and 1 mg/kg)-treated CIA mice exhibited repair of bone destruction compared with that in vehicle-treated CIA mice. TAS5315 exhibited dose-dependent therapeutic effects against decreases in BMD in the mouse CIA model, and TAS5315 (0.3 and 1 mg/kg) significantly improved declines in BMD compared to that in the vehicle-treated mice. In contrast, prednisolone tended to improve the decline in BMD but did not exhibit significant therapeutic effects (Fig 6C).

Finally, we assessed whether the bone quality was improved by TAS5315 in a CIA mouse model. The ultimate force and strain were used as parameters of the mechanical strength of bone. Analysis of the mechanical strength of tibiae in both hind limbs by diaphysis compression test revealed that TAS5315 dose-dependently improved the decreased ultimate force in CIA mice compared to that in normal mice (S7a Fig). Furthermore, TAS5315 improved the decline in ultimate strain in CIA mice (S7b Fig).

Therefore, these results suggest that TAS5315 represents an efficacious agent for RA by inhibiting bone destruction as well as joint inflammation in two rodent models of experimental arthritis.

## Discussion

BTK plays a pivotal role in the intracellular signaling pathways via BCR, FcγR, and RANK. *BTK* gene abnormalities such as mutation or loss lead to dysfunction of B cells, monocytes/macrophages, and osteoclasts [15, 19, 32]. Thus, BTK is a promising therapeutic molecular target for treating autoimmune diseases such as RA and systemic lupus erythematosus [21, 22]. TAS5315 is a potent and selective BTK inhibitor that suppresses the activation of B cells by inhibiting the BCR signaling pathway. TAS5315 also inhibited the production of inflammatory factors in IgG-stimulated monocytes/macrophages and the bone resorption activity of osteoclasts induced by RANKL stimulation.

In this study, TAS5315 dose-dependently decreased the histopathological scores for inflammation and joint destruction in an established CIA mouse model. Consistent with the previous results for BTK inhibitors [22, 23, 25, 26, 36], TAS5315 suppressed the functions of monocytes/macrophages and osteoclasts by modulating the FcγR and RANK signaling pathways, respectively. A CIA model in mice lacking the FcγR chain revealed a decreased infiltration of inflammatory cells into the pannus and alleviation of joint destruction compared to those expressing the FcγR chain [43]. In addition, RANKL-neutralizing antibody ameliorated bone loss at the site of inflammation in a mouse model of CIA [44]. These data suggest that FcγR and RANK signaling play pivotal roles in the pathogenesis of CIA. Therefore, selective BTK inhibitors such as TAS5315 are presumed to exhibit potent efficacy against inflammation and bone erosion in CIA models by inhibiting FcγR and RANK signaling.

TAS5315 inhibited BTK with an IC_50_ of 2.0 nM; of the 168 kinases tested at 100 nM, only 5 kinases (BLK, TEC, TXK, ITK, and BMX) were inhibited by TAS5315 at concentrations within 10-fold of the IC_50_ of BTK. BLK is a member of the SRC family of kinases specifically expressed in the B-cell lineage, and its gene has been linked to systemic lupus erythematosus susceptibility [45]. TEC, TXK, and ITK are members of the TEC family of kinases along with BTK, and these enzymes are mainly expressed in hematopoietic cells [46]. Similar to BTK, TEC is involved in the activation of the FcγR and RANK signaling pathways in macrophages and osteoclasts [15, 19]. ITK and TXK regulate the T-cell receptor signaling pathway and are involved in the activation and differentiation of T cells, respectively. The dual ITK/TXK inhibitor RN694 exhibited efficacious effects in animal models of immune diseases such as colitis and psoriasis [47, 48]. Thus, suppressing the activity of these kinases by TAS5315 might provide additional efficacy in terms of inhibiting effector cells associated with the pathogenesis of immune diseases, including RA.

Moreover, TAS5315 regulated the B-cell and T-cell phenotypes characterized by IgG secretion and by soluble IL-2 and IL-17A production, respectively, in the BT system of the BioMAP panel. Furthermore, TAS5315 suppressed the expression of CD86 and MHC class II induced by anti-IgM stimulation in mouse splenic B cells. These data indicate that TAS5315 may indirectly regulate T-cell activation by inhibiting the expression of T-cell co-stimulatory molecules via BCR signaling. Based on the results of the biochemical assays, TAS5315 might also regulate T-cell activation by directly inhibiting ITK and TXK. Additionally, TAS5315 may exhibit potent anti-inflammatory effects by directly or indirectly regulating the T cell function.

TAS5315 inhibited BTK phosphorylation and NFATc1 nuclear translocation in macrophages induced by RANKL; however, TAS5315 did not increase NFATc1 expression in cytosol fraction of RANKL-stimulated macrophages. The expression of NFATc1 is regulated by an auto-amplification loop during the process of osteoclastogenesis [49]. TAS5315 might modulate not only NFATc1 nuclear translocation but also the expression of NFATc1 by blocking the RANK-NFAT signaling pathway via BTK inhibition.

Several BTK inhibitors reportedly suppress the development of bone erosion in a mouse CIA model under a prophylactic treatment schedule [22, 23, 25–28, 36]. In the present study, TAS5315 repaired joint destruction in a mouse CIA model under a treatment schedule, as determined via a micro-CT analysis. Moreover, in an *in vitro* assay, TAS5315 suppressed the differentiation and bone resorption activity of osteoclasts induced by RANKL and M-CSF stimulation. The anti-human RANKL monoclonal antibody denosumab improved BMD and bone strength in ovariectomized cynomolgus monkeys [50] and inhibited bone erosion progression compared with placebo in clinical trials for RA [51]. These data suggest that regulating the RANKL/RANK signaling pathway via BTK inhibition may be effective against bone erosion in patients with RA.

Bone strength is related to BMD and bone quality. Declines in bone quality may pose a risk of fracture despite improvements in BMD caused by bisphosphonates [52, 53]. Here, TAS5315 improved the decline in ultimate force and ultimate strain in the tibias of an established CIA mouse model (S7A and S7B Fig). The ultimate force reflects the hardness of the bone, whereas the ultimate strain reflects its elasticity. These data indicate that TAS5315 can improve bone quality as well as BMD in patients with RA.

In clinical studies for patients with RA (the DRIVE study), denosumab inhibited the progression of bone erosion without revealing anti-inflammatory effects [51, 54]. In contrast, various DMARDs (methotrexate or inhibitors of TNFα or IL-6) have only limited effects on the progression of bone erosion [5]. In mouse models of CIA, TAS5315 inhibits inflammation (B cells, monocytes/macrophages) and bone damage (osteoclasts) and ameliorated synovitis and joint degeneration. These data suggest that TAS5315 can be used as a novel therapeutic agent for various symptoms of RA, such as inflammation, synovitis, and bone erosion. The safety and efficacy of TAS5315 are currently being evaluated in clinical trials for patients with RA, and we aim to provide RA patients with a new treatment alternative.

## Supporting information

S1 Text Additional methods for experiments in the supporting information. (DOCX)

S1 Fig Flow cytometry gating strategy for B220-positive B lymphocytes. Total lymphocytes were initially gated on an FSC versus SSC plot (left) and then gated on the CD45R/B220-positive B-cell population (center). Cell surface expressions of CD69, CD86, and I-A/I-E on CD45R/B220-positive B cells (right).
(TIF)

S2 Fig Kinase selectivity of TAS5315. (TIF)

S3 Fig Inhibitory effects of TAS5315 on multiple cell functions in BioMAP primary human multicellular systems. Biomarker readouts measured in each system are indicated along the x-axis. The y-axis depicts the log10 expression ratios of readout level measurements in the TAS5315-treated group (*n* = 1) relative to the control (DMSO-treated) group (*n* ≥ 6). The gray areas above and below the y-axis origin indicate the 95% significance envelope of the control group.
(TIF)

S4 Fig Direct and indirect effects of TAS5315 on monocytes/macrophages and FLS. (a) TNF-α levels in the culture supernatant of THP-1 cells stimulated with human IgG. (b, c) Levels of TNF-α and MIP-1α in the culture supernatant of BMDMs stimulated with mouse IgG. (d, e) Cell proliferation (d) and MMP-3 production in FLS (e) induced by TNF-α stimulation. Pretreatment with TAS5315 was performed for 30 min. (f, g) Human FLS proliferation (f) and MMP-3 production levels (g) in the presence of culture supernatants obtained from THP-1 cells treated with human IgG. TAS5315 treatment was performed simultaneously for each stimulation. Data are presented as the mean ± SD (*n* = 3–4 per group). *P<0.05, **P<0.01 compared with TNF-α or IgG plus DMSO group (S4A–S4C, S4F, S4G Fig: Dunnett test for TAS5315 groups, S4D, S4E Fig: Student’s *t*-test for TAS5315 group).
(TIF)

S5 Fig TAS5315 suppresses osteoclast differentiation and bone resorption activity. (a) Representative images of TRAP-stained osteoclasts. Osteoclast differentiation was induced by RANKL (66 ng/mL) and M-CSF under the condition of treatment with TAS5315 or DMSO for 4 days. (b) Representative images of pit formation by mouse osteoclasts. Osteoclasts were stimulated by RANKL (25 ng/mL) and M-CSF (50 ng/mL) under the treatment with TAS5315 or DMSO for 16 days.
(TIF)

S6 Fig TAS5315 suppresses the production of inflammatory cytokines in a mouse CIA model. (a) Arthritis score in the CIA mouse model on day 15. Experimental arthritis induced by two immunizations using collagen and CFA. Mice were administered vehicle or TAS5315 once daily for 14 days from 6th day after the second immunization. b) TNF-α, IL-1β, and IL-6 levels in synovial fluid exudates on day 15. Data are presented as the mean ± SEM (*n* = 6–8 per group). *P<0.05, **P<0.01, ***P<0.001 compared with vehicle group (Steel test).
(TIF)

S7 Fig Therapeutic effect of TAS5315 on the decrease in bone strength in a mouse CIA model. (a, b) Experimental arthritis induced by two immunizations with collagen and CFA. After therapeutic treatment with vehicle or TAS5315 once daily for 21 days from 12th day after the second immunization, mouse tibias were used to evaluate the mechanical bone strength. The vertical axis depicts values for ultimate force (the maximum force that the bone sustained) (a) and ultimate strain (pressed length until bone fracture) (b) as determined by diaphysis compression testing of the tibias of both hind limbs. Data are presented as the mean ± SEM (*n* = 8 per group). *P<0.05, compared with the vehicle group (Dunnett test for TAS5315 groups).
(TIF)

S1 Raw images Blot and gel images. (PDF)

S1 Table Pharmacokinetic parameters of rats orally administered with TAS5315. TAS5315 concentrations were measured in plasma at 0.5, 1, 2, 4, 6, 8, and 24 h after oral administration of TAS5315 at doses of 0.00127, 0.00381, 0.0127, 0.0381, 0.127, and 0.381 mg/kg (dosing volume of 10 mL/kg) (*n* = 3).
(XLSX)

S2 Table Components of the BioMAP panel. (XLSX)

S3 Table Numerical data of graphs in figures. (XLSX)
