# Supplementary material for: Novel Bruton’s tyrosine kinase inhibitor TAS5315 suppresses the progression of inflammation and joint destruction in rodent collagen-induced arthritis
Source: PLoS One. 2023 Feb 23;18(2):e0282117. doi: 10.1371/journal.pone.0282117 (PMC9949657; doi:10.1371/journal.pone.0282117)
Supplement: S1 Text — (DOCX) [file pone.0282117.s001.docx]

**Additional methods for experiments in the supplementary information**

- **Cell culture** All cells were cultured in an incubator at 37 °C and 5% CO_2_. THP-1 cells (ATCC, Manassas, VA, USA) were maintained in RPMI-1640 containing 10% fetal bovine serum (FBS) and 1% Pen-Strep (Pencillin-Streptomycin). Fibroblast-like synoviocytes (FLSs) derived from a patient with RA, collected with proper consent for all specimens, were purchased from Articular Engineering (Northbrook, IL, USA). FLSs derived from a patient with RA were maintained in Dulbecco’s modified Eagle’s medium (DMEM) containing 10% FBS and 1% Pen-Strep.
- **Compound profiling in the BioMAP panel** Cellular profiling of TAS5315 was performed using the BioMAP Diversity PLUS panel (DiscoverX, South San Francisco, CA, USA). Panel components (cell types, stimuli, and readouts) are summarized in the S2 Table.
   The systems are comprised of either single-cell types or co-culture systems. Adherent cell types were cultured in 96- or 384-well plates until confluence, followed by the addition of peripheral blood mononuclear cells (PBMCs) (SAg and LPS systems). Various concentrations of TAS5315 or dimethyl sulfoxide (DMSO) were added at the indicated concentrations 1 h before stimulation and were exposed to the culture medium for 24 h or as otherwise indicated (48 h, MyoF system; 72 h, BT system (soluble readouts); 168 h, BT system (secreted IgG)). The biomarker levels of cell-associated and cell membrane targets were measured using direct enzyme-linked immunosorbent (ELISA). Soluble factors from supernatants were quantified using either HTRF^®^ detection, bead-based multiplex immunoassay, or capture ELISA. Overt adverse effects of TAS5315 on cell proliferation and viability (cytotoxicity) were detected by sulforhodamine B (SRB) staining for adherent cells and alamarBlue staining for cells in suspension. For proliferation assays, individual cell types were cultured at subconfluence and were measured at time points optimized for each system (48 h: 3C and CASM3C systems; 72 h: BT and HDF3CGF systems; 96 h: SAg system). Cytotoxicity for adherent cells was measured by SRB staining (24 h: 3C, 4H, LPS, SAg, BF4T, BE3C, CASM3C, HDF3CGF, KF3CT, and lMphg systems; 48 h: MyoF system) and by alamarBlue staining for cells in suspension (24 h: SAg system; 42 h: BT system) at the time points indicated.
- **TNF-α and MIP-1α production by mouse macrophages and THP-1 cells.** Bone marrow-derived macrophages (BMDMs) were obtained as previously reported [1]. The BMDMs (1×10^4^ cells/well) in DMEM containing 10% FBS and 1% Pen-Strep were treated with TAS5315 or DMSO in an IgG-coated 96-well plate (Mouse IgG2A Isotype Control, R&D Systems; and Immulon 4 HBX 96-well plate, Thermo Fisher Scientific). The culture supernatant was collected after 4 h of incubation, and the levels of tumor necrosis factor (TNF)-α or macrophage inflammatory protein (MIP)-1α were determined using specific ELISAs (R&D Systems).
   THP-1 cells (1×10^5^ cells/well) were treated with TAS5315 or DMSO in an IgG-coated 96-well plate (Normal Human IgG Control; R&D Systems). The culture supernatant was collected after incubation for 4 h, and the TNF-α level was determined using specific ELISAs (R&D Systems).
- **FLS proliferation and MMP-3 production.** To prepare THP-1-conditioned medium, THP-1 cells (1×10^5^ cells/well) were treated with DMSO or TAS5315 in an IgG-coated 96-well plate and incubated for 4 h, after which the culture supernatant was collected.
   To assess cell proliferation, FLSs derived from patients with RA (5×10^4^ cells/well) were treated with TAS5315 and TNF-α (10 ng/mL) or with the THP-1-conditioned medium at a volume equal to that of the FLS medium. After 72 h of incubation, cell proliferation was assessed using a Cell Counting Kit 8 (Dojindo, Tokyo, Japan).
   For the matrix metalloproteinase (MMP)-3 production assay, FLSs derived from patients with RA (2×10^4^ cells/well) were treated with TAS5315 and TNF-α (10 ng/mL) or with the THP-1-conditioned medium at a volume equal to that of the FLS medium. After 24 h of incubation, the level of MMP-3 in the culture supernatant was determined by ELISA (R&D Systems).
- **Measurement of TNF-α, IL-1β, and IL-6 in synovial fluid exudates derived from hind paws of collagen-induced arthritis (CIA) mice.** Male DBA/1JNCrlj mice (8 weeks old) were injected with complete Freund's adjuvant (CFA) containing 2 mg/mL of bovine type II collagen at a site on the back (each 0.1 mL) and were boosted 21 days later in a similar manner. TAS5315 was administered orally for 14 consecutive days, starting from 6th day after the second immunization. Synovial fluid exudates for cytokine measurement were harvested the day after the final administration. Synovial fluid exudates were collected by lavaging the patella and around the joints with 0.3 mL of phosphate-buffered saline containing 1% bovine serum albumin. Levels of TNF-α, interleukin (IL)-1β, and IL-6 in synovial fluid exudates were determined by specific ELISAs (Thermo Fisher Scientific for TNF-α and Il-6; R&D Systems for IL-1β).
- **Mechanical strength test of the tibia in the CIA model** Male DBA/1JNCrlj mice (6 weeks old) were injected with CFA containing 2 mg/mL of bovine type II collagen at a site on the back (each 0.1 mL) and were boosted 21 days later in a similar manner. TAS5315 was administered orally for 14 consecutive days from 12th day after the second immunization. Mouse tibias for the mechanical strength test were harvested on the day after the final administration. The mechanical strength of the bone was measured by a compression test. A compressive load was applied to the proximal epiphysis of the tibia at a crosshead speed of 5 mm/min and a compression load of 500 N using a material-testing machine (MZ500D; Maruto Instrument Co., Tokyo, Japan). The parameters analyzed were ultimate force (N, maximum load value before bone broken) and ultimate strain (mm, distortion until bone broken). This experiment was carried out at the laboratory of Hamri Co., Ltd. (Ibaraki, Japan).

Reference

1. Park JK, Byun JY, Park JA, Kim YY, Lee YJ, Oh JI, et al. HM71224, a novel Bruton's tyrosine kinase inhibitor, suppresses B cell and monocyte activation and ameliorates arthritis in a mouse model: a potential drug for rheumatoid arthritis. Arthritis research & therapy. 2016;18:91. Epub 2015/01/01. doi: 10.1186/s13075-016-0988-z. PubMed PMID: 27090981; PubMed Central PMCID: PMC4835877.
