# Supplementary figures and images for: Novel Bruton’s tyrosine kinase inhibitor TAS5315 suppresses the progression of inflammation and joint destruction in rodent collagen-induced arthritis
Source: PLoS One. 2023 Feb 23;18(2):e0282117. doi: 10.1371/journal.pone.0282117 (PMC9949657; doi:10.1371/journal.pone.0282117)

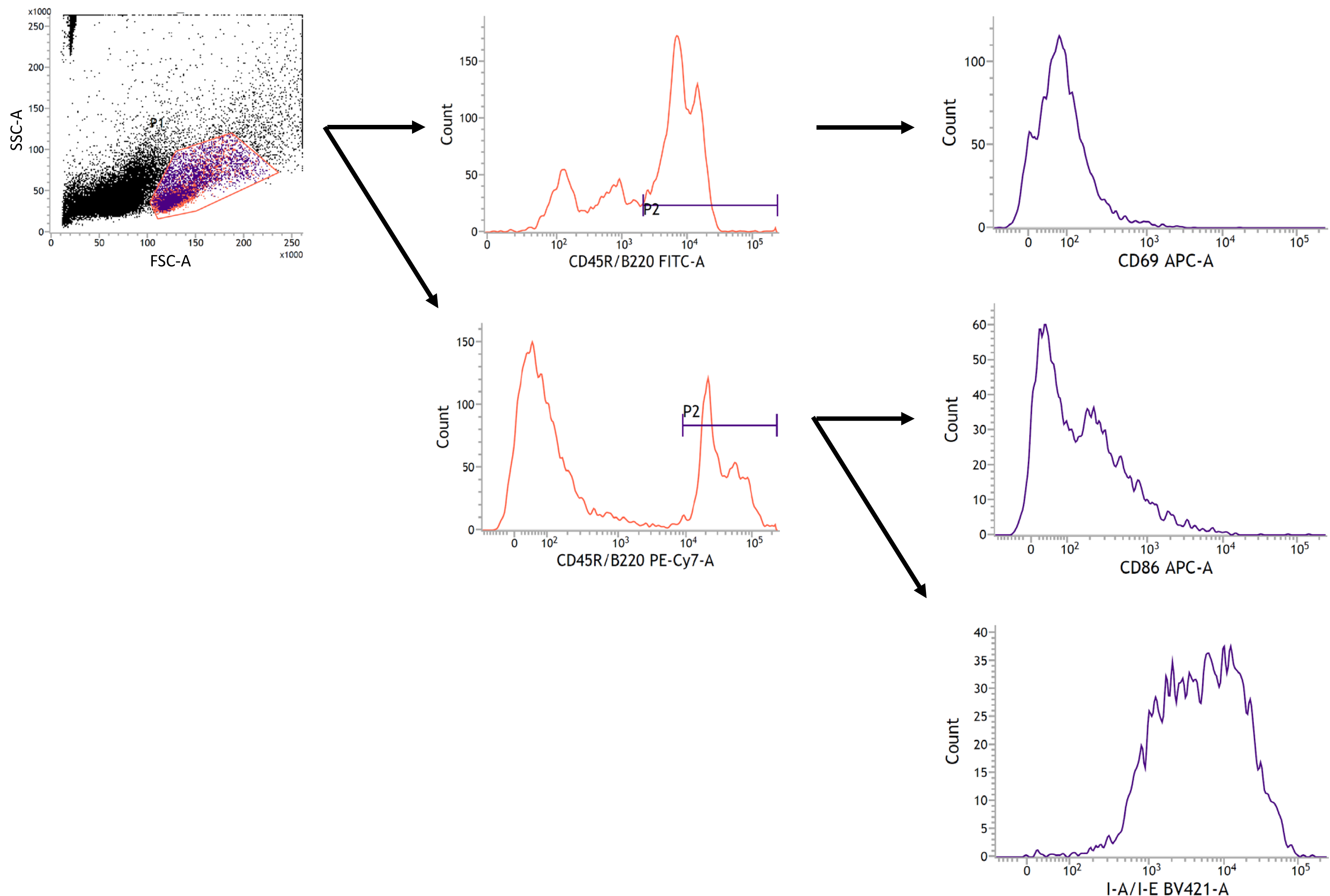

Supplement: S1 Fig — Total lymphocytes were initially gated on an FSC versus SSC plot (left) and then gated on the CD45R/B220-positive B-cell population (center). Cell surface expressions of CD69, CD86, and I-A/I-E on CD45R/B220-positive B cells (right). (TIF) [file pone.0282117.s002.tif]

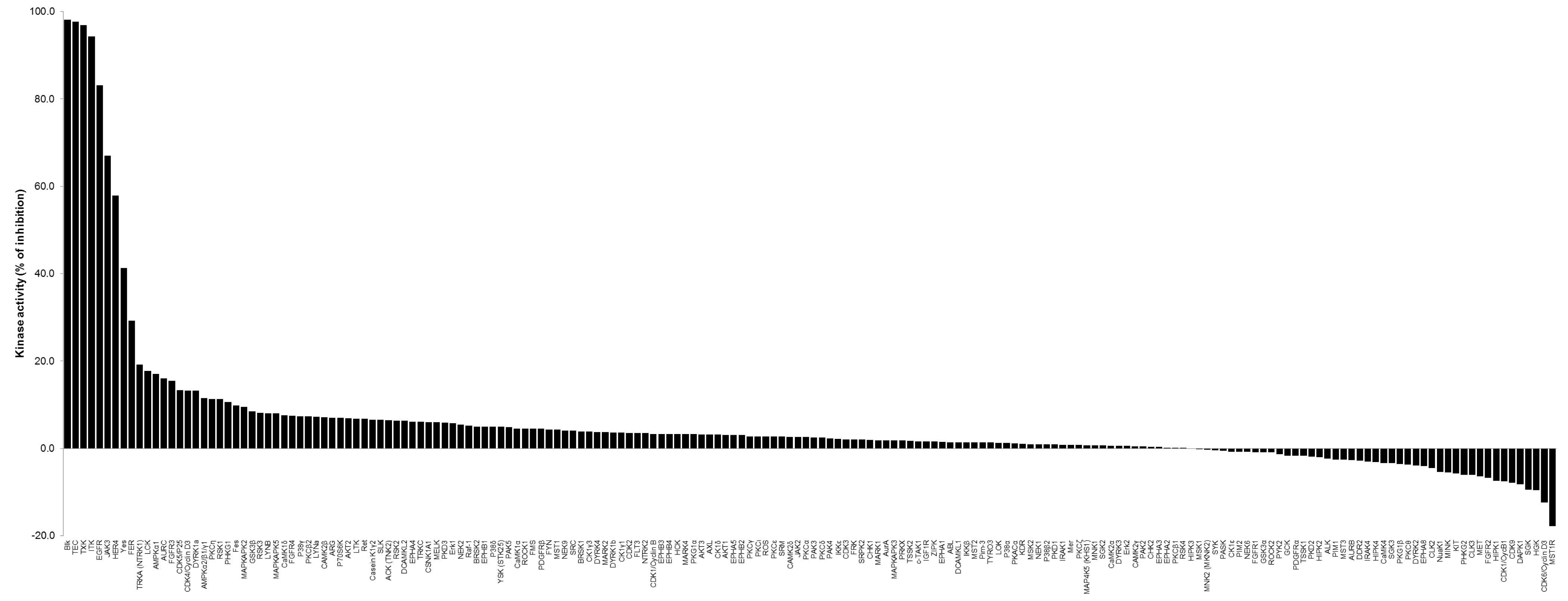

Supplement: S2 Fig — (TIF) [file pone.0282117.s003.tif]

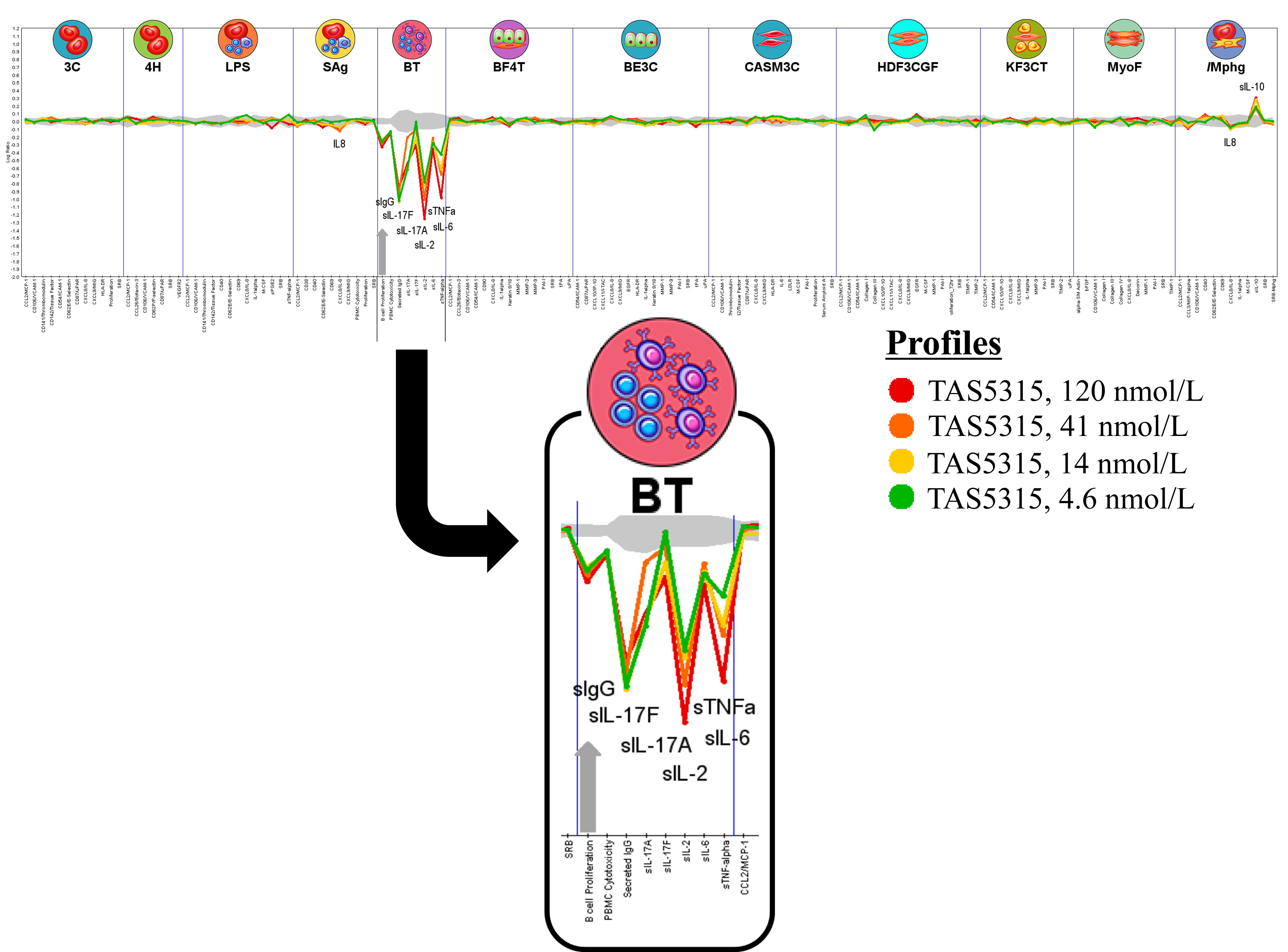

Supplement: S3 Fig — Biomarker readouts measured in each system are indicated along the x-axis. The y-axis depicts the log10 expression ratios of readout level measurements in the TAS5315-treated group (n = 1) relative to the control (DMSO-treated) group (n ≥ 6). The gray areas above and below the y-axis origin indicate the 95% significance envelope of the control group. (TIF) [file pone.0282117.s004.tif]

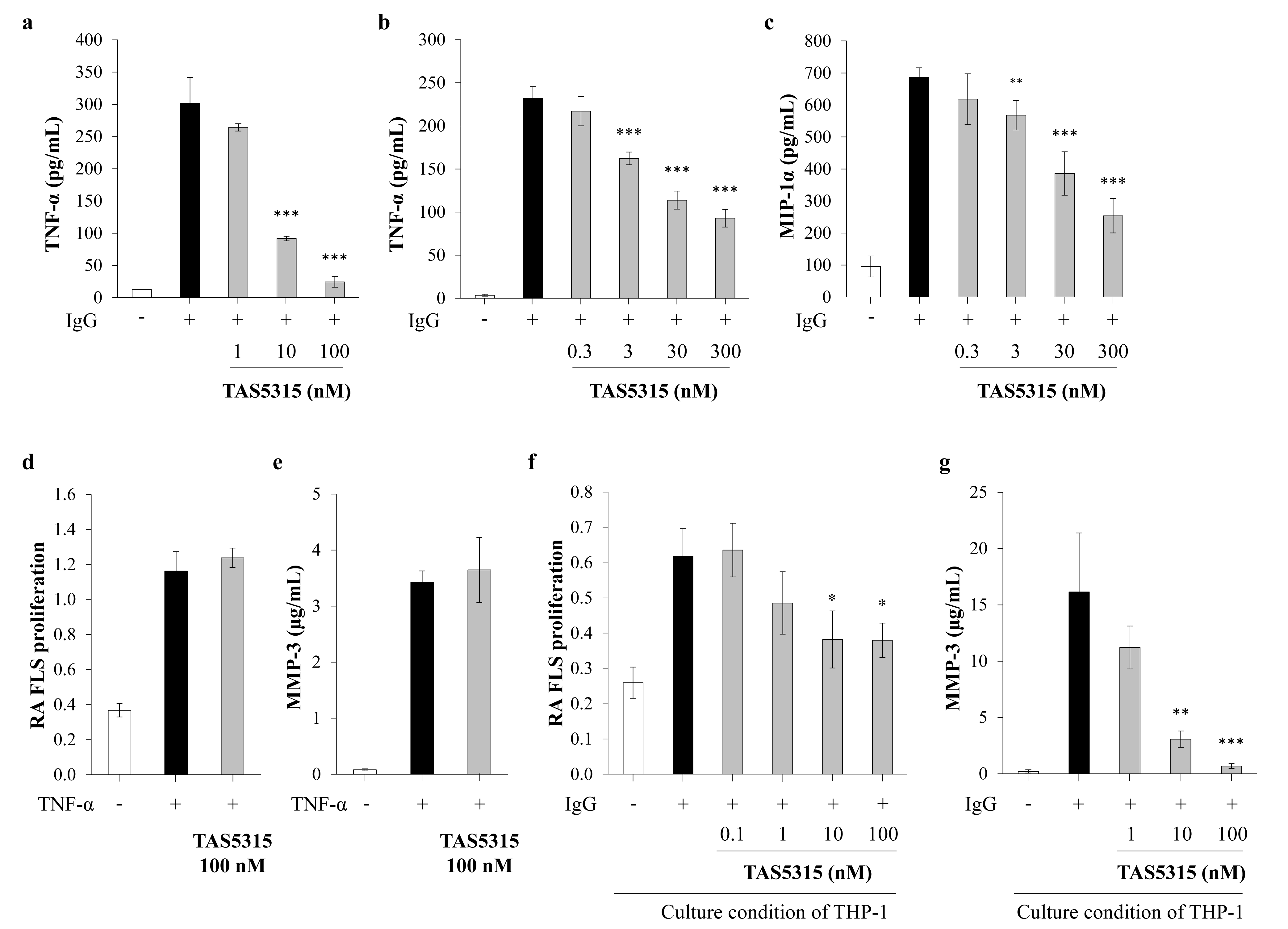

Supplement: S4 Fig — (a) TNF-α levels in the culture supernatant of THP-1 cells stimulated with human IgG. (b, c) Levels of TNF-α and MIP-1α in the culture supernatant of BMDMs stimulated with mouse IgG. (d, e) Cell proliferation (d) and MMP-3 production in FLS (e) induced by TNF-α stimulation. Pretreatment with TAS5315 was performed for 30 min. (f, g) Human FLS proliferation (f) and MMP-3 production levels (g) in the presence of culture supernatants obtained from THP-1 cells treated with human IgG. TAS5315 treatment was performed simultaneously for each stimulation. Data are presented as the mean ± SD (n = 3–4 per group). *P<0.05, **P<0.01 compared with TNF-α or IgG plus DMSO group (S4A–S4C, S4F, S4G Fig: Dunnett test for TAS5315 groups, S4D, S4E Fig: Student’s t-test for TAS5315 group). (TIF) [file pone.0282117.s005.tif]

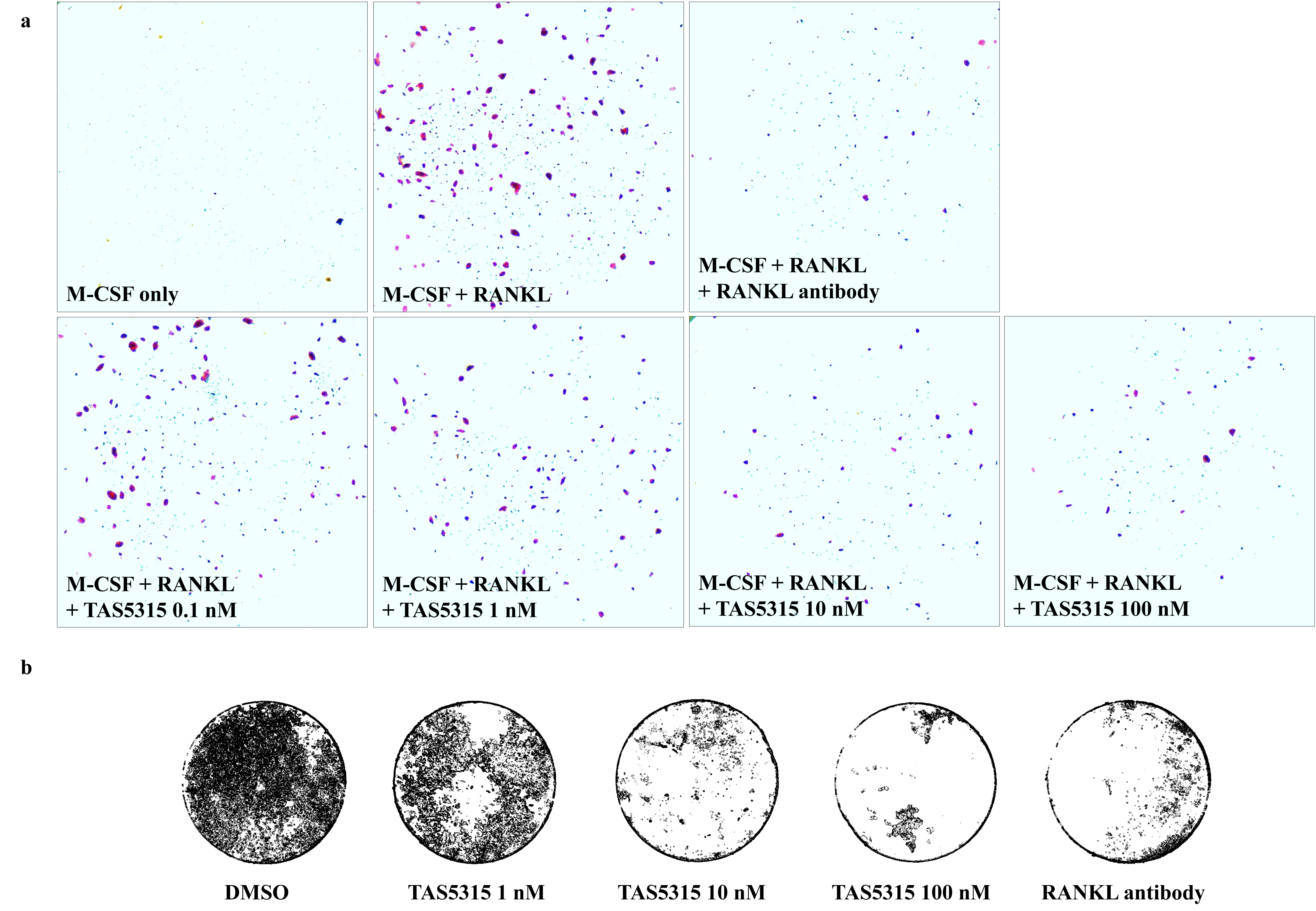

Supplement: S5 Fig — (a) Representative images of TRAP-stained osteoclasts. Osteoclast differentiation was induced by RANKL (66 ng/mL) and M-CSF under the condition of treatment with TAS5315 or DMSO for 4 days. (b) Representative images of pit formation by mouse osteoclasts. Osteoclasts were stimulated by RANKL (25 ng/mL) and M-CSF (50 ng/mL) under the treatment with TAS5315 or DMSO for 16 days. (TIF) [file pone.0282117.s006.tif]

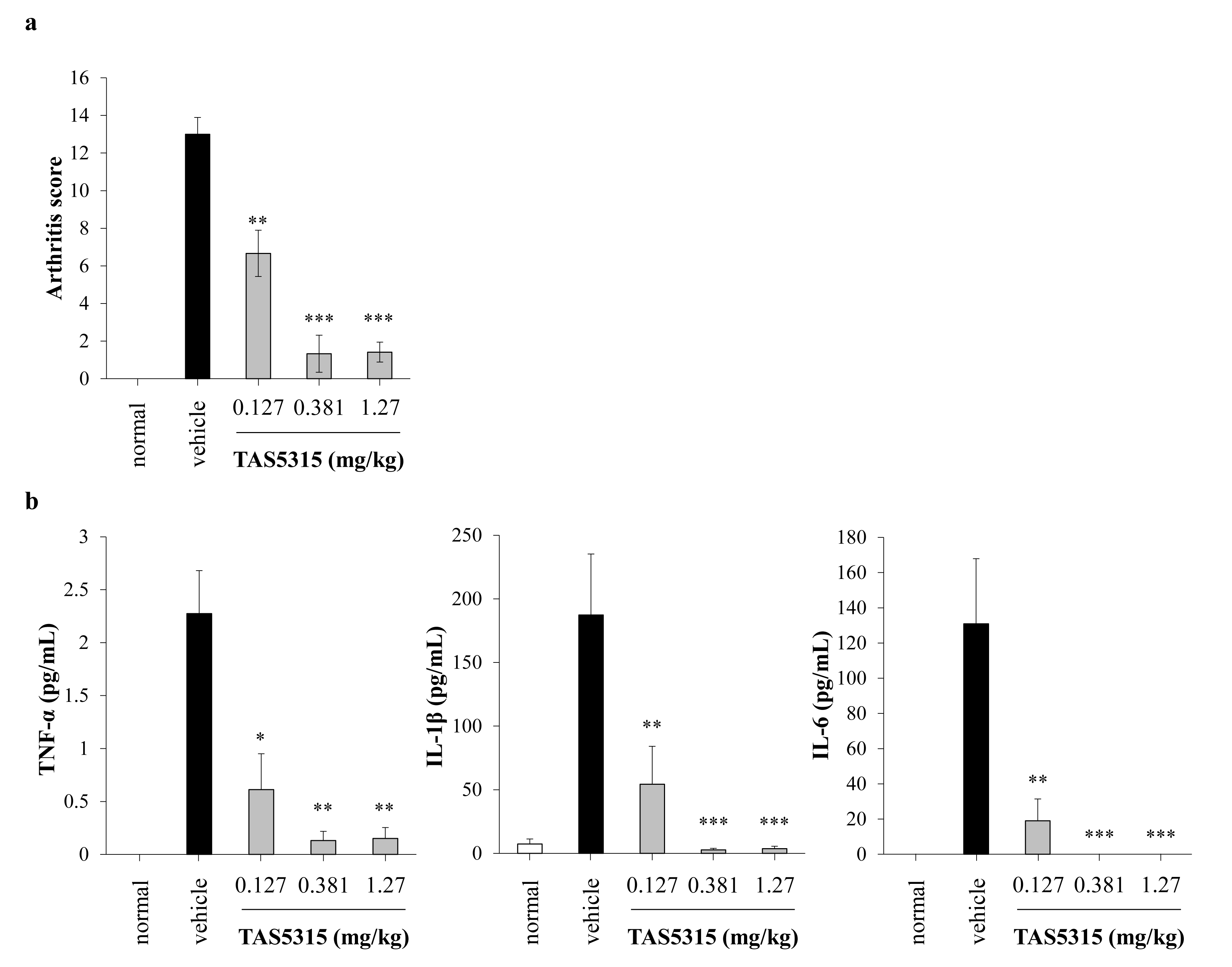

Supplement: S6 Fig — (a) Arthritis score in the CIA mouse model on day 15. Experimental arthritis induced by two immunizations using collagen and CFA. Mice were administered vehicle or TAS5315 once daily for 14 days from 6th day after the second immunization. b) TNF-α, IL-1β, and IL-6 levels in synovial fluid exudates on day 15. Data are presented as the mean ± SEM (n = 6–8 per group). *P<0.05, **P<0.01, ***P<0.001 compared with vehicle group (Steel test). (TIF) [file pone.0282117.s007.tif]

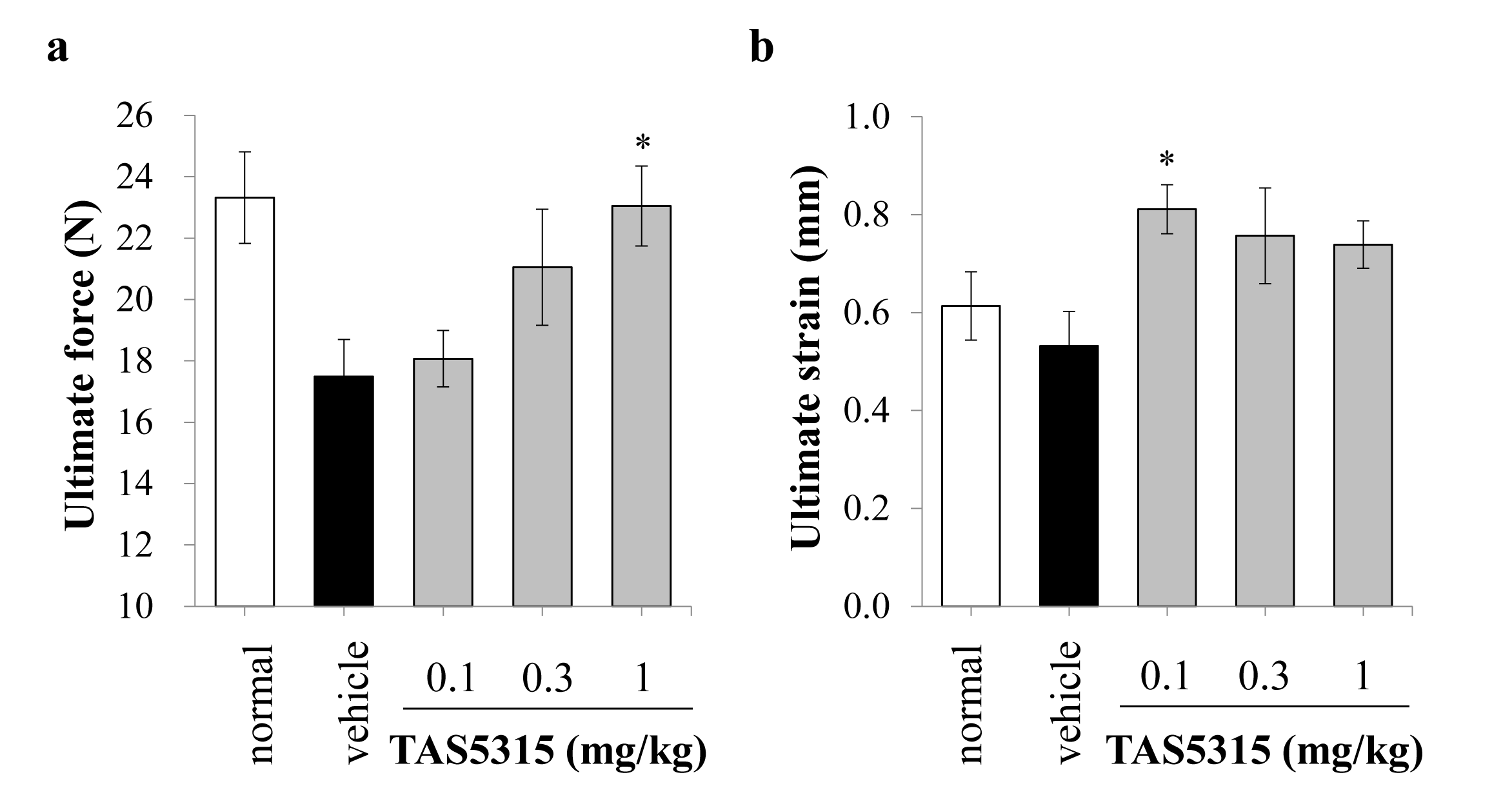

Supplement: S7 Fig — (a, b) Experimental arthritis induced by two immunizations with collagen and CFA. After therapeutic treatment with vehicle or TAS5315 once daily for 21 days from 12th day after the second immunization, mouse tibias were used to evaluate the mechanical bone strength. The vertical axis depicts values for ultimate force (the maximum force that the bone sustained) (a) and ultimate strain (pressed length until bone fracture) (b) as determined by diaphysis compression testing of the tibias of both hind limbs. Data are presented as the mean ± SEM (n = 8 per group). *P<0.05, compared with the vehicle group (Dunnett test for TAS5315 groups). (TIF) [file pone.0282117.s008.tif]
