# Supplementary material for: Novel Bruton’s tyrosine kinase inhibitor TAS5315 suppresses the progression of inflammation and joint destruction in rodent collagen-induced arthritis
Source: PLoS One. 2023 Feb 23;18(2):e0282117. doi: 10.1371/journal.pone.0282117 (PMC9949657; doi:10.1371/journal.pone.0282117)
Supplement: S1 Raw images — (PDF) [file pone.0282117.s009.pdf]

# Fig 1a_phosphorylated BTK

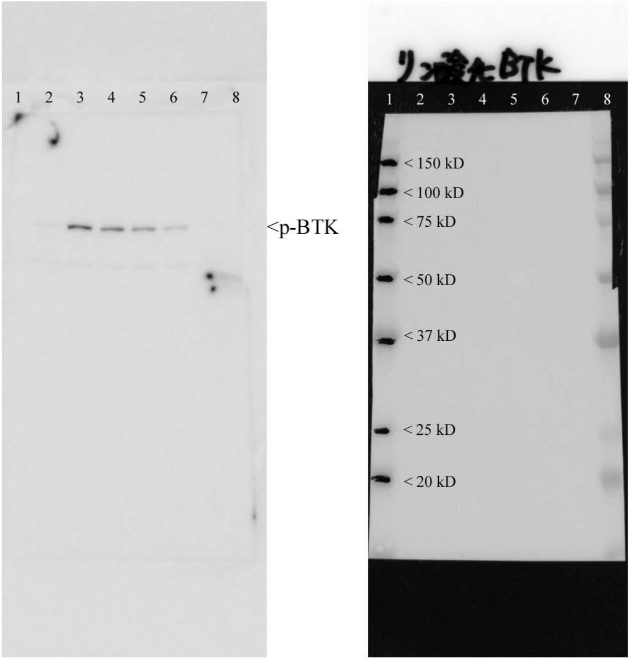


1. molecular weight marker
2. - α-IgM, DMSO
3. + α-IgM, DMSO
4. + α-IgM, TAS5315 0.03 nM
5. + α-IgM, TAS5315 0.1 nM
6. + α-IgM, TAS5315 0.3 nM
7. + α-IgM, TAS5315 1 nM
8. molecular weight marker

# Fig 1a_BTK

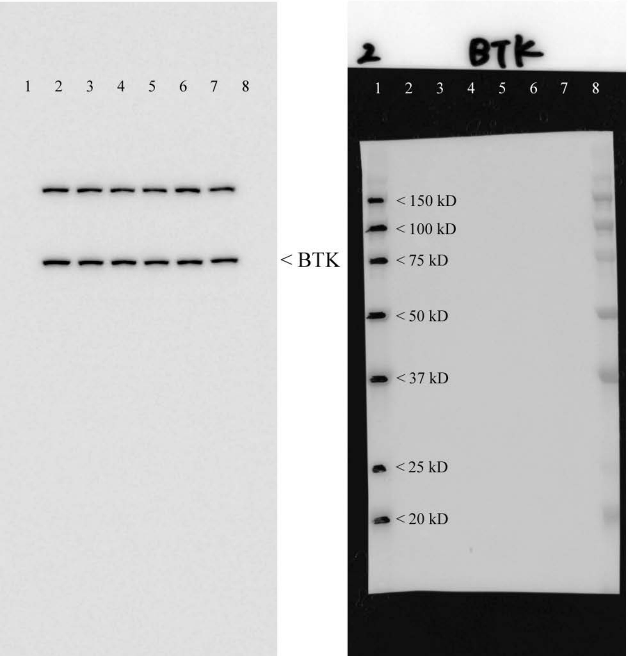


1. molecular weight marker
2. - α-IgM, DMSO
3. + α-IgM, DMSO
4. + α-IgM, TAS5315 0.03 nM
5. + α-IgM, TAS5315 0.1 nM
6. + α-IgM, TAS5315 0.3 nM
7. + α-IgM, TAS5315 1 nM
8. molecular weight marker

# Fig 1a_G3PDH

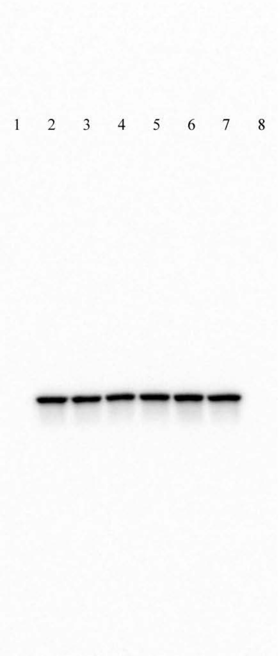


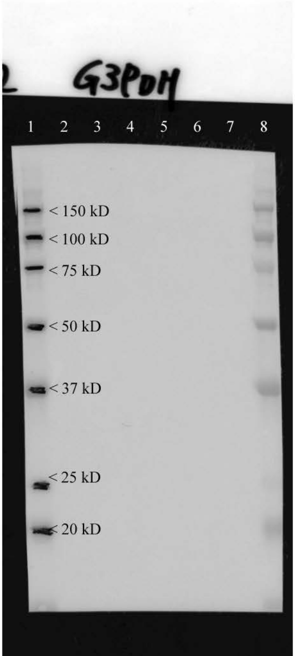


1. molecular weight marker
2. - α-IgM, DMSO
3. + α-IgM, DMSO
4. + α-IgM, TAS5315 0.03 nM
5. + α-IgM, TAS5315 0.1 nM
6. + α-IgM, TAS5315 0.3 nM
7. + α-IgM, TAS5315 1 nM
8. molecular weight marker

# Fig 1c_phosphorylated BTK

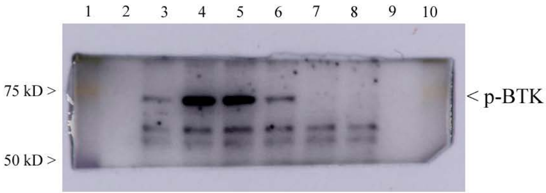


1. molecular weight marker
2. blank well
3. - α-IgM, DMSO
4. + α-IgM, DMSO
5. + α-IgM, TAS5315 0.01 nM
6. + α-IgM, TAS5315 0.1 nM
7. + α-IgM, TAS5315 1 nM
8. + α-IgM, TAS5315 10 nM
9. blank well
10. molecular weight marker

# Fig 1c_BTK

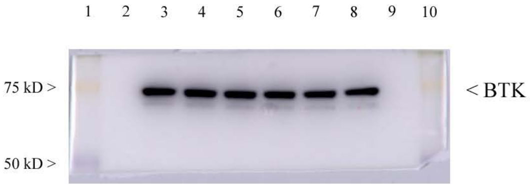


1. molecular weight marker
2. blank well
3. - α-IgM, DMSO
4. + α-IgM, DMSO
5. + α-IgM, TAS5315 0.01 nM
6. + α-IgM, TAS5315 0.1 nM
7. + α-IgM, TAS5315 1 nM
8. + α-IgM, TAS5315 10 nM
9. blank well
10. molecular weight marker

# Fig 1c_phophorylated PLCγ2

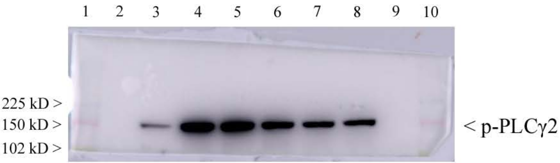


1. molecular weight marker
2. blank well
3. - α-IgM, DMSO
4. + α-IgM, DMSO
5. + α-IgM, TAS5315 0.01 nM
6. + α-IgM, TAS5315 0.1 nM
7. + α-IgM, TAS5315 1 nM
8. + α-IgM, TAS5315 10 nM
9. blank well
10. molecular weight marker

# Fig 1c_PLCγ2

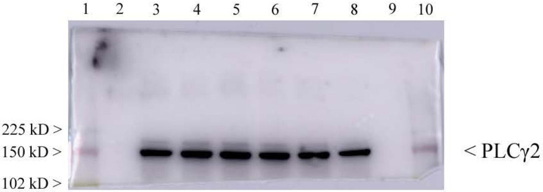


1. molecular weight marker
2. blank well
3. - α-IgM, DMSO
4. + α-IgM, DMSO
5. + α-IgM, TAS5315 0.01 nM
6. + α-IgM, TAS5315 0.1 nM
7. + α-IgM, TAS5315 1 nM
8. + α-IgM, TAS5315 10 nM
9. blank well
10. molecular weight marker

# Fig 1c_phosphorylated AKT

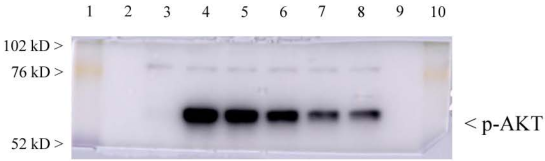


1. molecular weight marker
2. blank well
3. - α-IgM, DMSO
4. + α-IgM, DMSO
5. + α-IgM, TAS5315 0.01 nM
6. + α-IgM, TAS5315 0.1 nM
7. + α-IgM, TAS5315 1 nM
8. + α-IgM, TAS5315 10 nM
9. blank well
10. molecular weight marker

# Fig 1c_AKT

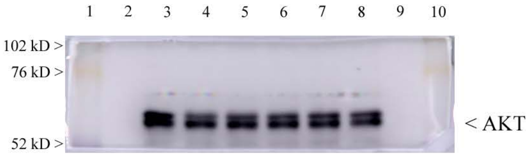


1. molecular weight marker
2. blank well
3. - α-IgM, DMSO
4. + α-IgM, DMSO
5. + α-IgM, TAS5315 0.01 nM
6. + α-IgM, TAS5315 0.1 nM
7. + α-IgM, TAS5315 1 nM
8. + α-IgM, TAS5315 10 nM
9. blank well
10. molecular weight marker

# Fig 1c_phosphorylated ERK

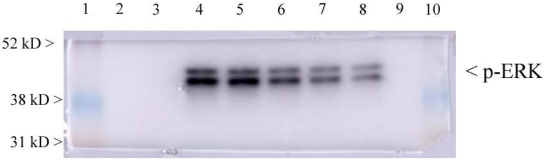


1. molecular weight marker
2. blank well
3. - α-IgM, DMSO
4. + α-IgM, DMSO
5. + α-IgM, TAS5315 0.01 nM
6. + α-IgM, TAS5315 0.1 nM
7. + α-IgM, TAS5315 1 nM
8. + α-IgM, TAS5315 10 nM
9. blank well
10. molecular weight marker

# Fig 1c_ERK

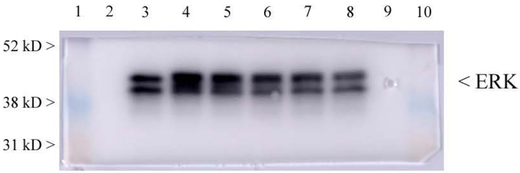


1. molecular weight marker
2. blank well
3. - α-IgM, DMSO
4. + α-IgM, DMSO
5. + α-IgM, TAS5315 0.01 nM
6. + α-IgM, TAS5315 0.1 nM
7. + α-IgM, TAS5315 1 nM
8. + α-IgM, TAS5315 10 nM
9. blank well
10. molecular weight marker

# Fig 1c_G3PDH

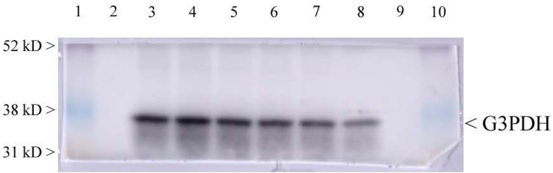


1. molecular weight marker
2. blank well
3. - α-IgM, DMSO
4. + α-IgM, DMSO
5. + α-IgM, TAS5315 0.01 nM
6. + α-IgM, TAS5315 0.1 nM
7. + α-IgM, TAS5315 1 nM
8. + α-IgM, TAS5315 10 nM
9. blank well
10. molecular weight marker

# Fig 3a_phosphorylated BTK

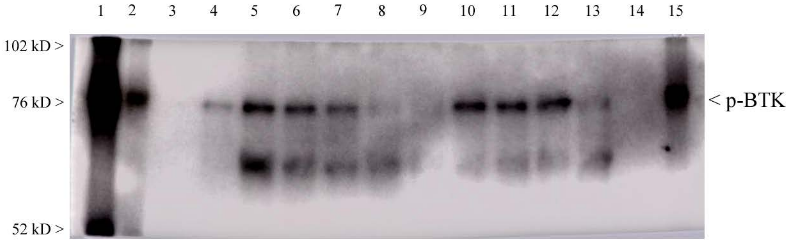


1. molecular weight marker
2. molecular weight marker
3. blank well
4. - RANKL, DMSO
5. + RANKL, DMSO
6. + RANKL, TAS5315 0.01 nM
7. + RANKL, TAS5315 0.1 nM
8. + RANKL, TAS5315 1 nM
9. + RANKL, TAS5315 10 nM

10~13. ×

14. blank well
15. molecular weight marker

# Fig 3a_BTK

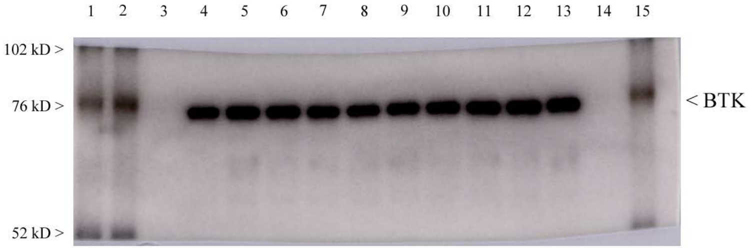


1. molecular weight marker
2. molecular weight marker
3. blank well
4. - RANKL, DMSO
5. + RANKL, DMSO
6. + RANKL, TAS5315 0.01 nM
7. + RANKL, TAS5315 0.1 nM
8. + RANKL, TAS5315 1 nM
9. + RANKL, TAS5315 10 nM

10~13. ×

14. blank well
15. molecular weight marker

# Fig 3a_G3PDH

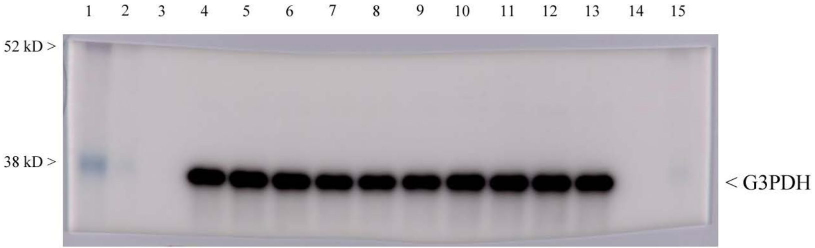


1. molecular weight marker
2. molecular weight marker
3. blank well
4. - RANKL, DMSO
5. + RANKL, DMSO
6. + RANKL, TAS5315 0.01 nM
7. + RANKL, TAS5315 0.1 nM
8. + RANKL, TAS5315 1 nM
9. + RANKL, TAS5315 10 nM

10~13. ×

14. blank well
15. molecular weight marker

# Fig 3b_NFATc1 in cytosol fraction

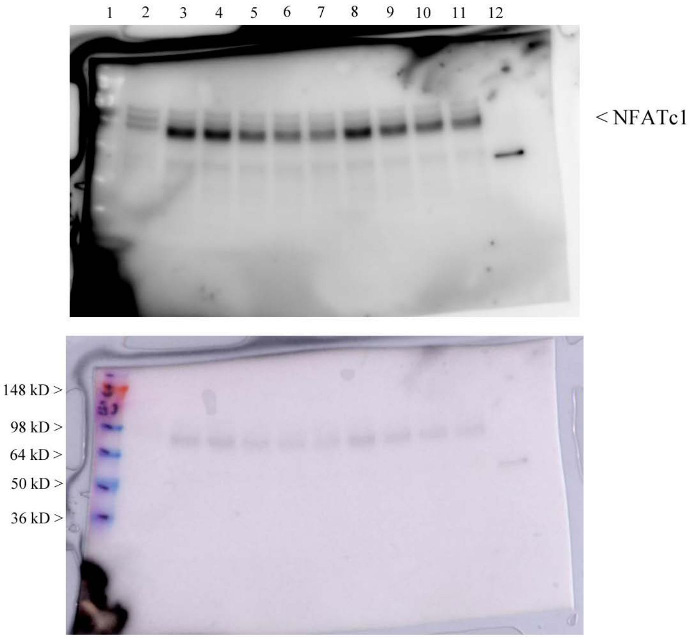


1. molecular weight marker
2. - RANKL, DMSO
3. + RANKL, DMSO
4. + RANKL, TAS5315 0.01 nM
5. + RANKL, TAS5315 0.1 nM
6. + RANKL, TAS5315 1 nM
7. + RANKL, TAS5315 10 nM

8~12. ×

# Fig 3b_NFATc1 in nuclear fraction

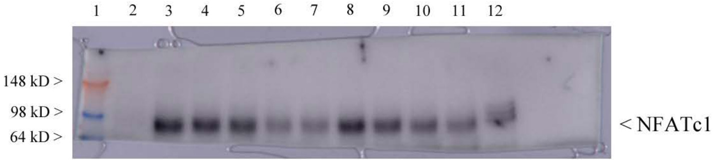


1. molecular weight marker
2. - RANKL, DMSO
3. + RANKL, DMSO
4. + RANKL, TAS5315 0.01 nM
5. + RANKL, TAS5315 0.1 nM
6. + RANKL, TAS5315 1 nM
7. + RANKL, TAS5315 10 nM

8~12. ×

# Fig 3b_p84 in cytosol fraction

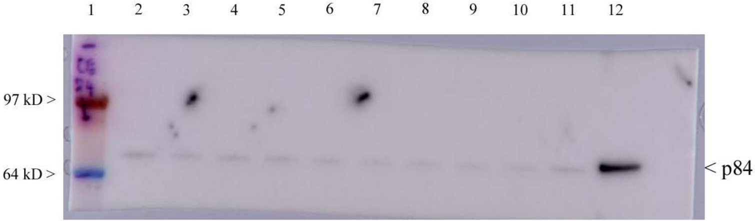


1. molecular weight marker
2. - RANKL, DMSO
3. + RANKL, DMSO
4. + RANKL, TAS5315 0.01 nM
5. + RANKL, TAS5315 0.1 nM
6. + RANKL, TAS5315 1 nM
7. + RANKL, TAS5315 10 nM

8~11. ×

12. nuclear fraction

# Fig 3b_p84 in nuclear fraction

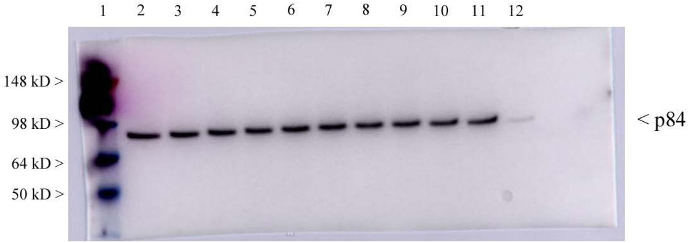


1. molecular weight marker
2. - RANKL, DMSO
3. + RANKL, DMSO
4. + RANKL, TAS5315 0.01 nM
5. + RANKL, TAS5315 0.1 nM
6. + RANKL, TAS5315 1 nM
7. + RANKL, TAS5315 10 nM

8~11. ×

12. cytosol fraction

# Fig 3b_β-tubulin in cytosol fraction

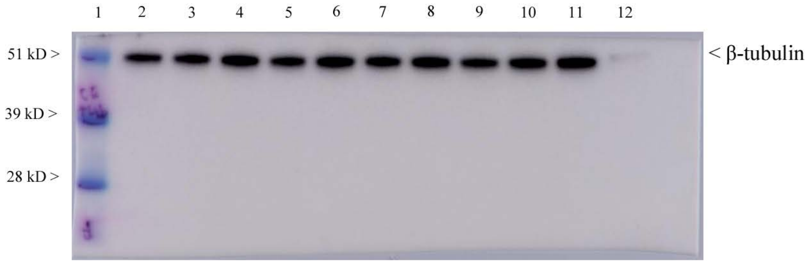


1. molecular weight marker
2. - RANKL, DMSO
3. + RANKL, DMSO
4. + RANKL, TAS5315 0.01 nM
5. + RANKL, TAS5315 0.1 nM
6. + RANKL, TAS5315 1 nM
7. + RANKL, TAS5315 10 nM

8~11. ×

12. nuclear fraction

# Fig 3b_β-tubulin in nuclear fraction

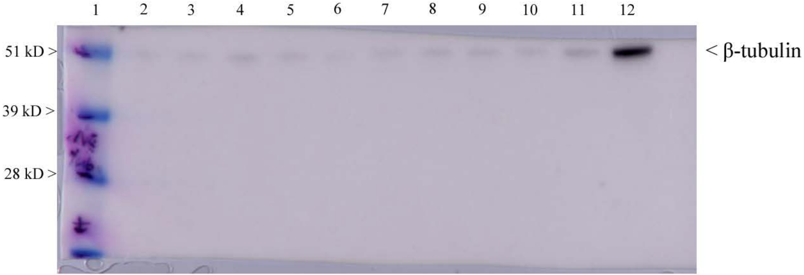


1. molecular weight marker
2. - RANKL, DMSO
3. + RANKL, DMSO
4. + RANKL, TAS5315 0.01 nM
5. + RANKL, TAS5315 0.1 nM
6. + RANKL, TAS5315 1 nM
7. + RANKL, TAS5315 10 nM

8~11. ×

12. cytosol fraction

# Fig 4a_occupied BTK

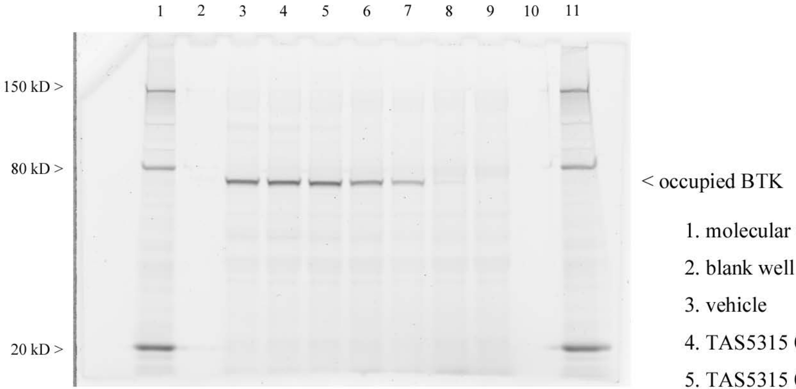


1. molecular weight marker
2. blank well
3. vehicle
4. TAS5315 0.00127 mg/kg
5. TAS5315 0.00381 mg/kg
6. TAS5315 0.0127 mg/kg
7. TAS5315 0.0381 mg/kg
8. TAS5315 0.127 mg/kg
9. TAS5315 0.381 mg/kg
10. blank well
11. molecular weight marker

# Fig 4a_BTK

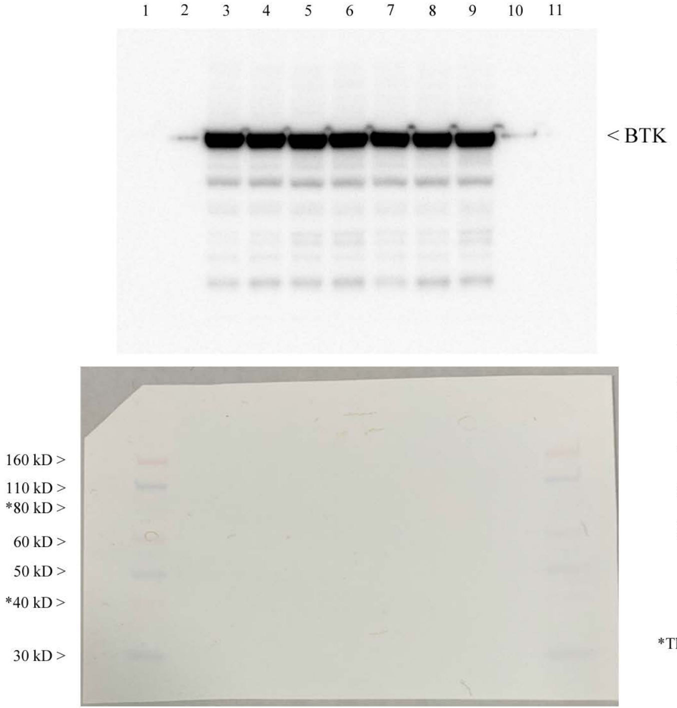


1. molecular weight marker
2. blank well
3. vehicle
4. TAS5315 0.00127 mg/kg
5. TAS5315 0.00381 mg/kg
6. TAS5315 0.0127 mg/kg
7. TAS5315 0.0381 mg/kg
8. TAS5315 0.127 mg/kg
9. TAS5315 0.381 mg/kg
10. blank well
11. molecular weight marker

*The red and orange band corresponding to respectively 80 and 40 kD have faded due to aging of the membrane.
